# Complementary Inhibitory Weight Profiles Emerge from Plasticity and Allow Flexible Switching of Receptive Fields

**DOI:** 10.1523/JNEUROSCI.0276-20.2020

**Published:** 2020-12-09

**Authors:** Everton J. Agnes, Andrea I. Luppi, Tim P. Vogels

**Affiliations:** ^1^Centre for Neural Circuits and Behaviour, University of Oxford, Oxford, OX1 3SR, United Kingdom; ^2^Institute of Science and Technology Austria, Klosterneuburg, 3400, Austria

**Keywords:** cortex, disinhibition, EI balance, receptive field, synaptic plasticity

## Abstract

Cortical areas comprise multiple types of inhibitory interneurons, with stereotypical connectivity motifs that may follow specific plasticity rules. Yet, their combined effect on postsynaptic dynamics has been largely unexplored. Here, we analyze the response of a single postsynaptic model neuron receiving tuned excitatory connections alongside inhibition from two plastic populations. Synapses from each inhibitory population change according to distinct plasticity rules. We tested different combinations of three rules: Hebbian, anti-Hebbian, and homeostatic scaling. Depending on the inhibitory plasticity rule, synapses become unspecific (flat), anticorrelated to, or correlated with excitatory synapses. Crucially, the neuron's receptive field (i.e., its response to presynaptic stimuli) depends on the modulatory state of inhibition. When both inhibitory populations are active, inhibition balances excitation, resulting in uncorrelated postsynaptic responses regardless of the inhibitory tuning profiles. Modulating the activity of a given inhibitory population produces strong correlations to either preferred or nonpreferred inputs, in line with recent experimental findings that show dramatic context-dependent changes of neurons' receptive fields. We thus confirm that a neuron's receptive field does not follow directly from the weight profiles of its presynaptic afferents. Our results show how plasticity rules in various cell types can interact to shape cortical circuit motifs and their dynamics.

**SIGNIFICANCE STATEMENT** Neurons in sensory areas of the cortex are known to respond to specific features of a given input (e.g., specific sound frequencies), but recent experimental studies show that such responses (i.e., their receptive fields) depend on context. Inspired by the cortical connectivity, we built models of excitatory and inhibitory inputs onto a single neuron, to study how receptive fields may change on short and long time scales. We show how various synaptic plasticity rules allow for the emergence of diverse connectivity profiles and, moreover, how their dynamic interaction creates a mechanism by which postsynaptic responses can quickly change. Our work emphasizes multiple roles of inhibition in cortical processing and provides a first mechanistic model for flexible receptive fields.

## Introduction

Inhibitory neurons exhibit large variability in morphology, connectivity motifs, and electrophysiological properties (Markram et al., 2004; Jiang et al., 2015; Jouhanneau et al., 2018; Gouwens et al., 2019). Inhibition often balances excitatory inputs, thus stabilizing neuronal network activity (Van Vreeswijk and Sompolinsky, 1996; Vogels et al., 2011) and allowing for a range of different functions (Hennequin et al., 2017; Maffei, 2017; Sprekeler, 2017; Chiu et al., 2019; Nicola and Clopath, 2019). When both inhibitory and excitatory inputs share the same statistics and their weight profiles are similar (Froemke et al., 2007), the resulting state of the postsynaptic neuron is one of precise balance of input currents (Hennequin et al., 2017). Modulation of inhibition (e.g., a decrease or increase in local inhibitory activity) and, consequently, a change in the balance between excitation and inhibition, can control the activity of neuronal groups (Letzkus et al., 2015; Kuchibhotla et al., 2017), and it is believed that disinhibition is an important mechanism for the implementation of high-level brain functions, such as attention (Zhang et al., 2014; Kuchibhotla et al., 2017), memory retrieval (Vogels et al., 2011; Barron et al., 2016; Vallentin et al., 2016), signal gating (Vogels and Abbott, 2009; Kremkow et al., 2010), and rapid learning (Nicola and Clopath, 2019).

The state of balance is thought to be achieved and maintained by inhibitory plasticity, for example, a Hebbian-like inhibitory plasticity rule (Vogels et al., 2011) (increase in synaptic weights for correlated presynaptic and postsynaptic activity), as observed in auditory cortex (D'amour and Froemke, 2015). Other types of inhibitory plasticity have also been observed, for example, changes in chloride reversal potential (Woodin et al., 2003) that locally decrease the driving force of inhibitory synapses following correlated presynaptic and postsynaptic activity, suggesting a form of anti-Hebbian inhibitory plasticity that has been proposed as a mechanism for memory formation (Hendin et al., 1997).

Given that cortical circuit motifs feature multiple interneuron types (Jiang et al., 2015; Maffei, 2017; Gouwens et al., 2019), we wondered how these opposing types of plasticity may act in concert on the same postsynaptic target, and how the resulting synaptic weight profiles can help to shape the receptive field (i.e., the neurons' response to presynaptic stimuli). We speculated that two plasticity rules could form complementary synaptic weight profiles for inhibitory connections, such that synapses following a Hebbian-like inhibitory plasticity rule would mirror excitatory inputs; anti-Hebbian plasticity should impose strong inhibitory inputs for weak excitatory ones, and vice versa. Such opposite wiring profiles of distinct inhibitory synapse populations are in line with intracellular recordings showing that strong inhibitory postsynaptic potentials can be elicited by stimuli with preferred orientations of the postsynaptic neuron (Ferster, 1986; Douglas and Martin, 1991), but also by stimuli with nonpreferred orientations (Volgushev et al., 1993; Pei et al., 1994). What's more, dynamically changing receptive fields could be achieved through targeted modulation of a specific type of inhibition.

Altered receptive field properties have been widely observed (e.g., in mouse auditory cortex where neurons change their preferred sound frequency with varying sound intensity) (Bathellier et al., 2012). In macaque primary visual cortex (V1), neurons can modulate their response according to an extra cue of a different (auditory) sensory nature (McClure and Polack, 2019). Intriguingly, they responded either more strongly to their preferred stimulus or, on the contrary, they were more suppressed when a pure tone was played alongside the presentation of the visual stimuli. In macaque V4 (Ruff and Cohen, 2014) and V5 (Ruff and Cohen, 2019), neurons have been shown to change how they represent different stimuli during detection and discrimination tasks; and in macaque V4 (Benjamin et al., 2019), some neurons change their hue preference when subjected to single-hue or naturally colored images. Receptive field profiles have also been shown to induce localized changes (around preferred inputs) in attention tasks (Fritz et al., 2003), and adaptation of the postsynaptic activity to repetitive stimulation (Kohn and Movshon, 2004). Finally, recent work by Billeh et al. (2019) showed that, in mice, visual neurons change their response to the direction of motion of visual stimuli depending on either the temporal or the spatial frequency of the stimulus (drifting grating). These results suggest that receptive fields of sensory neurons are dramatically affected by input (i.e., contextual/attentional states) (Fritz et al., 2003; Kohn and Movshon, 2004; Ruff and Cohen, 2014, 2019; Benjamin et al., 2019; McClure and Polack, 2019), or different aspects of the sensory stimulus (Bathellier et al., 2012; Billeh et al., 2019), but it is unclear by which mechanisms such changes can transpire.

**Figure 1.**
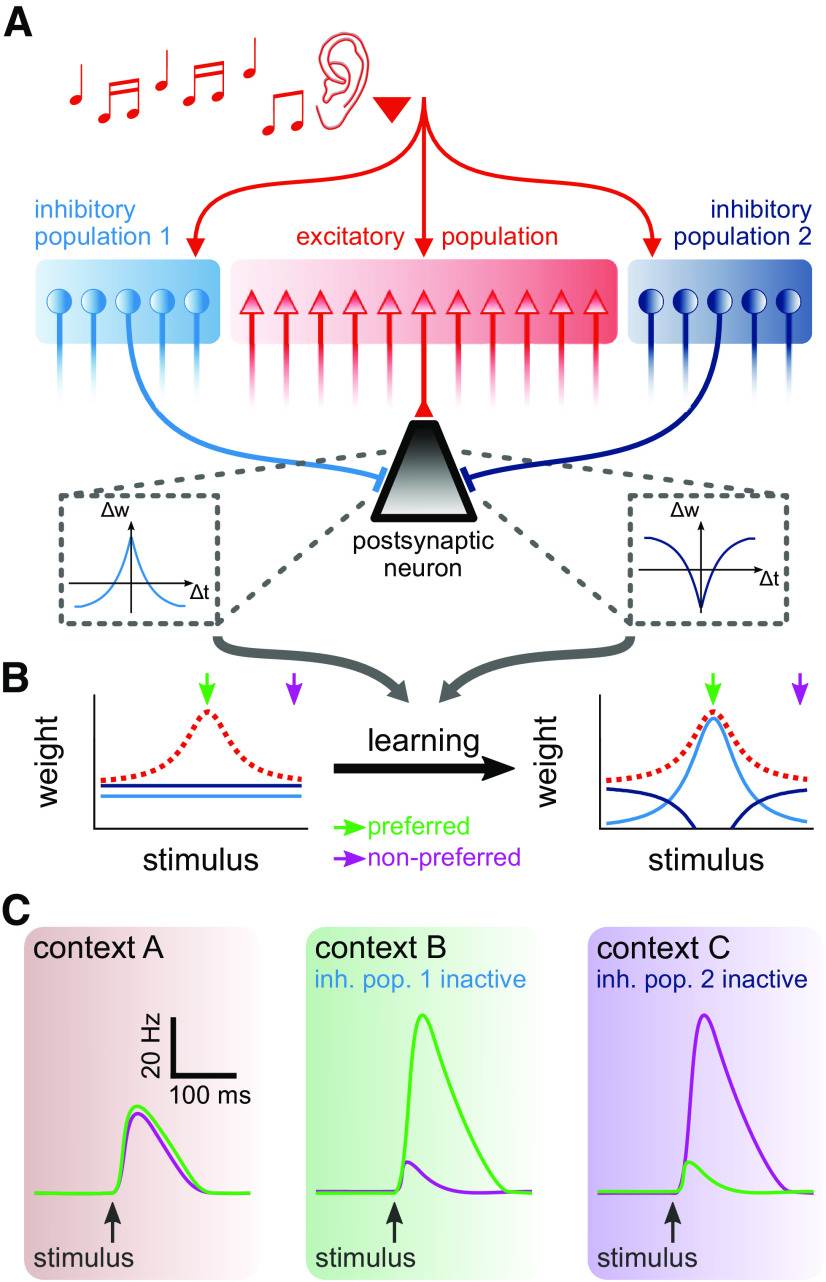
Learning of two distinct inhibitory populations and postsynaptic response because of attentional switch between contexts. ***A***, Schematic of co-active plasticity rules. A postsynaptic neuron (black triangle) receives tuned excitatory input (red population) and inhibition from two distinct populations (blue populations). The two inhibitory populations follow different synaptic plasticity rules. Δ*w* indicates change in synaptic weight, and Δ*t* indicates interval between presynaptic and postsynaptic spikes. ***B***, Initially untuned inhibitory weights (blue lines) acquire different synaptic weight profiles after learning that depend on the excitatory weight profile (red dashed line). ***C***, Contextual changes (e.g., because of attention), which we hypothesize to be responsible for modulating the activity of inhibitory populations, result in different postsynaptic responses to the same stimulus (Bathellier et al., 2012; Ruff and Cohen, 2014, 2019; Benjamin et al., 2019; Billeh et al., 2019; McClure and Polack, 2019), such that preferred (green) and nonpreferred (purple) stimuli elicit postsynaptic responses with different amplitudes.

Here, we tested how the response of a single neuron is affected when the activity of presynaptic inhibitory populations is modulated. We combined two hypotheses to address this question. First, we considered that different types of inhibitory interneurons may follow distinct synaptic plasticity learning rules (Fig. 1*A*), thus creating different connectivity profiles onto postsynaptic neurons (Hennequin et al., 2017) (Fig. 1*B*), such as those observed for parvalbumin-positive (PV^+^) and somatostatin-positive (SOM^+^) interneurons (Wilson et al., 2012). PV^+^ interneurons may follow a Hebbian-like plasticity rule (Vogels et al., 2011; D'amour and Froemke, 2015), thus targeting pyramidal neurons with similar preferred orientation (Wilson et al., 2012). SOM^+^ interneurons, on the other hand, could follow a non-Hebbian plasticity rule (e.g., anti-Hebbian or homeostatic), which results in a nonselective connectivity (Wilson et al., 2012). Our second hypothesis posits that changes in the activity of inhibitory neurons are responsible for the highly variable receptive fields observed in recent experiments (Bathellier et al., 2012; Ruff and Cohen, 2014, 2019; Benjamin et al., 2019; Billeh et al., 2019; McClure and Polack, 2019) (Fig. 1*C*). This hypothesis extrapolates from evidence of cortical disinhibition during functional tasks (Pi et al., 2013; Letzkus et al., 2015), and requires that a different brain region provide attentional or contextual signals, such as observed in PFC and regions in the frontal lobe (Noudoost et al., 2010; Anderson et al., 2011; Baluch and Itti, 2011; Benchenane et al., 2011).

To determine the origins of such varying responses from the same cell, we investigated the behavior of a single postsynaptic neuron model receiving tuned excitatory inputs, and inhibition from two distinct populations. Input tuning may correspond to preference for a specific sound frequency (Froemke et al., 2007), orientation of visual cues (Smith et al., 2013), or to color hue (Benjamin et al., 2019), taste (Haley et al., 2016), whisker stimulation (Estebanez et al., 2018), or position in space (Harvey et al., 2009). We show that, when distinct biologically plausible plasticity rules operate on the synapses of different inhibitory populations, at least three different tuning profiles may emerge. After learning, the postsynaptic neuron arrives at a balanced state with respect to its excitatory and inhibitory inputs. In this state, preferred signals are transiently revealed, but steady-state responses are indiscriminate of the stimulus preference (Vogels et al., 2011) (i.e., its “orientation,” etc.), regardless of the inhibitory connectivity. However, we could substantially alter the responses of the postsynaptic neuron by modulating the activity of either of the two presynaptic inhibitory populations, allowing for the propagation of facets of the input patterns that were previously quenched by inhibition. Such inhibitory modulation can thus serve as a mechanism to selectively filter stimuli according to, for example, attentional cues, as observed in recent experiments (Bathellier et al., 2012; Ruff and Cohen, 2014, 2019; Benjamin et al., 2019; Billeh et al., 2019; McClure and Polack, 2019). In summary, our work proposes a simple biological implementation for an attentional switch of input selectivity, and provides a solution for how such a neuronal circuit can emerge with autonomous and unsupervised, biologically plausible plasticity rules. To our best knowledge, our model is the first proof of principle that the receptive field of a neuron (i.e., its response to presynaptic stimuli) does not have to follow directly from the (excitatory) presynaptic weight profiles.

## Materials and Methods

### 

#### Neuron model

To investigate changes in neuronal response because of specific inhibitory connectivity motif, we simulated a postsynaptic leaky integrate-and-fire neuron (LIF) receiving excitatory and inhibitory afferents. Postsynaptic neuronal membrane potential dynamics is governed by the following:

$$\tau_{\text{m}}\frac{\text{d}u(t)}{\text{d}t}=-\left[u(t)-u_{\text{rest}}\right]$$

(1) $$-g_{\text{E}}(t)\left[u(t)-E_{\text{E}}\right]-g_{\text{I}}(t)\left[u(t)-E_{\text{I}}\right],$$

where *u*(*t*) is the somatic voltage at time *t*, τ_m_ = *RC* is the membrane time constant (membrane resistance, *R*, times membrane capacitance, *C*), *u*_rest_ is the resting membrane potential, and *E*_E_ and *E*_I_ are the reversal potential for excitatory and inhibitory synapses, respectively. Synaptic conductances, *g*_E_ (*t*) and *g*_I_ (*t*), evolve according to the following:

(2) $$\frac{\text{d}g_{\text{E}}(t)}{\text{d}t}=-\frac{g_{\text{E}}(t)}{\tau_{\text{E}}}+\sum_{j=1}^{N_{\text{E}}}w_{j}(t)S_{j}(t)$$

and

(3) $$\frac{\text{d}g_{\text{I}}(t)}{\text{d}t}=-\frac{g_{\text{I}}(t)}{\tau_{\text{I}}}+\sum_{j=N_{\text{E}}+1}^{N}w_{j}(t)S_{j}(t).$$

Both excitatory and inhibitory conductances decay exponentially to zero with time constants τ_E_ and τ_I,_, respectively. Presynaptic action potentials trigger increase in synaptic conductances through the sum of Dirac δ functions as follows:

(4) $$S_{j}(t)=\sum_{k}\delta \left(t-t_{kj}\right),$$

where *t_kj_* is the time of the *k*th spike of presynaptic afferent *j*. The contribution of a given presynaptic afferent *j* to changes in conductances is given by the synaptic weight $w_{j}(t)$, which was fixed for excitatory synapses and could change over time because of plasticity mechanisms for inhibitory synapses. The total number of presynaptic afferents is $N=N_{\text{E}}+N_{\text{I}}$, with *N*_E_ being the number of excitatory and *N*_I_ of inhibitory presynaptic afferents.

An action potential is triggered at the postsynaptic neuron once its membrane potential *u*(*t*) crosses the spiking threshold *u*_th_ from below. The membrane potential is then instantaneously reset to *u*_reset,_ being clamped at this value for the duration of the refractory period, τ_ref._ The postsynaptic spike train is described as a sum of Dirac deltas as follows:

(5) $$S_{\text{post}}(t)=\sum_{k}\delta (t-t_{k}),$$

where *t_k_* is the time of the *k*th spike of the postsynaptic neuron, or the time the membrane potential crosses the spiking threshold from below. Parameters used for the postsynaptic neuron are detailed in Table 1.

**Table 1.** Simulation parameters for the postsynaptic neuron

Parameter	Symbol	Value	Figures
Membrane time constant	τ_m_	30 ms	4–11
Resting potential	*u*_rest_	–65 mV	4–11
Excitatory reversal potential	*E*_E_	0 mV	4–11
Inhibitory reversal potential	*E*_I_	–80 mV	4–11
Excitatory time constant	τ_E_	5 ms	4–11
Inhibitory time constant	τ_I_	10 ms	4–11
Spiking threshold	*u*_th_	–50 mV	4–11
Reset potential	*u*_reset_	–65 mV	4–11
Refractory period	τ_ref_	5 ms	4–11
Simulation time step	Δ*t*	0.1 ms	4–11

#### Inputs

To mimic experimentally observed synaptic input profiles (Froemke et al., 2007), we divided the synaptic inputs into *P* signal groups (μ = 1, …, *P*) that share the same fluctuation in firing rate. We tested two cases: natural input and pulse input. Both are described below.

##### Natural input

For presynaptic activity mimicking a natural input, activity follows an inhomogeneous Poisson process that changes according to a modified Ornstein-Uhlenbeck (OU) process. We first defined an auxiliary variable for each pattern, $y_{\mu }(t)$, that follows a stochastic first-order differential equation given by the following:

(6) $$\frac{\text{d}y_{\mu }(t)}{\text{d}t}=-\frac{y_{\mu }(t)}{\tau_{\text{OU}}}+\xi_{\mu }(t),$$

where μ is the signal group index, τ_OU_ is the time constant for the decaying process that changes because of a Gaussian noise term ξ_μ_(*t*) with unitary standard deviation (SD). The mean value of the variable *y*_μ_ is zero, and thus it assumes positive and negative values for long periods.

The spike train of an afferent in a given signal group μ is given by the variable, $\nu_{X\mu }(t)$, which is a rectified version of the auxiliary variable plus a term to generate background firing rate, $\nu_{X\text{bg}}$, where *X* indicates the presynaptic population (*X* = E for excitatory and *X* = I for inhibitory). The spike trains of the afferents of signal group μ are generated by the following:

(7) $$\nu_{X\mu }(t)=\nu_{X0}{\left[y_{\mu }(t)\right]}_{+}+\nu_{X\text{bg}},$$

where ν*_X_*_0_ is the amplitude of the modulated firing rate fluctuations, and ${\left[\cdot \right]}_{+}$ is a rectifying function, as follows:

(8) $${\left[y\right]}_{+}=\{\begin{array}{l} y,\text{ if }y>0\\ 0,\text{otherwise}. \end{array}$$

Because of the symmetry of *y*_μ_(*t*), an afferent is half the time in the background state and half the time in the active state.

Presynaptic action potentials were generated as an inhomogeneous Poisson process according to the modified OU process described above and a fixed background firing rate. Additionally, we implemented a refractory period, τ_Eref_ for excitatory and τ_Iref_ for inhibitory inputs. Given the time step of the simulation Δ*t*, spikes of a presynaptic afferent that is part of the signal group μ are generated with a probability $p_{X\mu }(t)=\nu_{X\mu }(t)\Delta t$ if there was no spike elicited during the refractory period beforehand, and thus the average firing rate of a *X* = E (excitatory) or *X* = I (inhibitory) afferent that is part of the signal group μ becomes the following:

(9) $$F_{X\mu }(t)=\frac{1}{\Delta t}p_{X\mu }(t){\left(1-p_{X\mu }(t)\right)}^{\tau_{X\text{ref}}/\Delta t}.$$

##### Pulse input

To test transient responses to brief changes in presynaptic activity, we also quantified postsynaptic responses to pulse inputs. In this case, we simulated the postsynaptic neuron receiving inputs with constant background firing rate. For 100 ms, we increased the probability of presynaptic spikes for a given signal group μ by a factor $k\nu^{*}$, with *k* being an integer larger or equal than zero and ν* = 5 Hz. Thus, presynaptic spikes are generated by the following:

(10) $$\nu_{X\mu }(t)=\alpha_{X\nu }k\nu^{*}+\nu_{X\text{bg}},$$

during the 100 ms step and by the following:

(11) $$\nu_{X\mu }(t)=\nu_{X\text{bg}}$$

during only background activity. Parameter α*_Xν_* is a scalar that sets the ratio of excitatory and inhibitory firing rate.

Responses to the pulse input were divided in two bins: phasic and tonic. Phasic responses were defined as the postsynaptic activity elicited in the first 50 ms of the pulse input. Tonic activity was correspondingly defined as having occurred in the last 50 ms of the stimulus. We simulated 100 trials per input strength $k\nu^{*}$, and defined the response (for both phasic and tonic) as the average number of spikes on the period for the strength $k\nu^{*}$ minus the average number of spikes on the same period without extra input, multiplied by 20 to convert to Hz. We subtracted background spikes to ascertain that we quantified the response to the extra step input alone. The tonic input window was used to assess persistent, steady-state postsynaptic responses to elevated inputs. To confirm that the firing behavior within these 50 ms indeed reflected a steady state, we ran control simulations with a variety of intervals: from 50 to 500 ms. All tests showed that our 50 ms window was sufficient to obtain a representative sample of steady-state, “tonic” activity. Parameters used for inputs are detailed in Table 2.

**Table 2.** Simulation parameters for the inputs

Parameter	Symbol	Value	Figures
No. of excitatory afferents	*N*_E_	3200	4–11
No. of inhibitory afferents	*N*_I_	800	4–11
No. of signal groups	*P*	16	4–11
Refractory period for excitatory afferents	τ_Eref_	5 ms	2, 4–11
Refractory period for inhibitory afferents	τ_Iref_	2.5 ms	2, 4–11
OU time constant	τ_OU_	50 ms	2, 4–11
OU update time step	Δ*T*	1 ms	2, 4–11
Excitatory firing rate amplitude for OU	ν_E0_	5 Hz	2, 4–11
Inhibitory firing rate amplitude for OU	ν_I0_	10 Hz	2, 4–11
Excitatory background firing rate	ν_Ebg_	2 Hz	2, 4–11
Inhibitory background firing rate	ν_Ibg_	4 Hz	2, 4–11
Pulse amplitude reference	ν*	5 Hz	4, 8, 10, 11
Excitatory ratio for pulse input	$\alpha_{\text{E}\nu }$	1	4, 8, 10, 11
Inhibitory ratio for pulse input	$\alpha_{\text{I}\nu }$	2	4, 8, 10, 11
Synaptic weight profile amplitude	*r*_0_	4	4–11
Synaptic weight profile slope	*b*	0.25	4–11
Preferred pattern index	μ_0_	9	4–11
Synaptic weight profile power	*c*	2	4–11
Simulation time step	Δ*t*	0.1 ms	2, 4–11

#### Synaptic tuning

Based on Vogels et al. (2011), we used a synaptic weight profile for the excitatory population given by the following:

(12) $$r(\mu )=\left(\frac{1}{1+r_{0}}\right)+\left(\frac{r_{0}}{1+r_{0}}\right)\left(\frac{1}{1+b{\left(\mu -\mu_{0}\right)}^{c}}\right),$$

where *r*_0_, *b*, and *c* are parameters defining the shape of the synaptic weight profile and μ_0_ defines the preferred signal group, which maximizes *r*(μ); *r*(μ_0_) = 1. Note that $r_{0}\geq 1,0<b\leq 1,\mu_{0}>0$, and *c* is an even positive integer.

For simplicity, we define ζ*_j_* as the signal group of which that afferent *j* is part. Thus, excitatory synapses are set as follows:

(13) $$w_{j}=w_{\text{E}0}r(\zeta_{j})+\epsilon_{j},\;j=1,...,N_{\text{E}},$$

where *w*_E0_ is a normalization factor for excitatory weights, and ϵ*_j_* is a noise term drawn from a uniform random distribution between $-\epsilon_{\text{E}}^{*}$ and $\epsilon_{\text{E}}^{*}$.

Initial conditions for all inhibitory populations were flat with noise (see Figs. 5, 7, 9), as follows:

(14) $$w_{j}(0)=w_{\text{IF}}+\epsilon_{j},\;j=N_{\text{E}}+1,...,N.$$

In all cases, except for one (see Fig. 5), all inhibitory synapses changed according to inhibitory plasticity rules (details below). For Figure 5, one population of inhibitory afferents were plastic and another was kept fixed throughout the learning period of the simulations. Parameters used for synaptic weights are detailed in Tables 2 and 3.

**Table 3.** Simulation parameters for the weights

Parameter	Symbol	Value	Figures
Excitatory baseline weight	*w*_E0_	0.5	4–11
Noise parameter for excitatory weights	$\epsilon_{\text{E}}^{*}$	0.01	4–11
Inhibitory baseline weight (one inhibitory population)	*w*_IF_	0.4	Data not shown
Noise parameter for inhibitory weights (one inhibitory population)	$\epsilon_{\text{I}}^{*}$	0.01	Data not shown
Inhibitory baseline weight	*w*_IF_	Varying	5, 6
Noise parameter for inhibitory weights	$\epsilon_{\text{I}}^{*}$	0.01	5, 6
Inhibitory baseline weight (Hebbian & scaling)	*w*_IF_	0.8	7
Noise parameter for inhibitory weights (Hebbian & scaling)	$\epsilon_{\text{I}}^{*}$	0.3	7
Inhibitory baseline weight (Hebbian & anti-Hebbian)	*w*_IF_	0.55	9
Noise parameter for inhibitory weights (Hebbian & anti-Hebbian)	$\epsilon_{\text{I}}^{*}$	0.01	9
Correcting factor for plot	α_w_	4.4	5, 7, 9

Because of the small number of inhibitory afferents compared with the excitatory ones, and the difference in driving force, inhibitory weights were much larger than excitatory ones. Thus, we plotted excitatory weights multiplied by the parameter α*_w_*.

#### Plasticity models

In this work, we used three different inhibitory synaptic plasticity rules. We termed them “Hebbian,” “scaling,” and “anti-Hebbian.” Both Hebbian and anti-Hebbian plasticity rules are triggered by presynaptic and postsynaptic spikes, and depend on a low-pass filter of these spike trains. The presynaptic trace (low-pass filter) is given by the following:

(15) $$\frac{\text{d}x_{j}(t)}{\text{d}t}=-\frac{x_{j}(t)}{\tau_{\text{STDP}}}+S_{j}(t),$$

where *x_j_*(*t*) is the value of the trace of the spike train of presynaptic afferent *j* at time *t*; τ_STDP_ is the time constant of the trace, and *S_j_*(*t*) is a sum of Dirac δ functions (Eq. 4) representing the spike train of afferent *j*. The same is considered for the postsynaptic neuron, as follows:

(16) $$\frac{\text{d}x_{\text{post}}(t)}{\text{d}t}=-\frac{x_{\text{post}}(t)}{\tau_{\text{STDP}}}+S_{\text{post}}(t),$$

where *x*_post_ (*t*) is the postsynaptic trace, and *S*_post_ (*t*) is the spike train of the postsynaptic neuron (Eq. 5). We used the same time constant for both presynaptic and postsynaptic traces.

##### Hebbian inhibitory plasticity

Precise balance of excitatory and inhibitory inputs was learned by a Hebbian inhibitory plasticity rule (Vogels et al., 2011). The weight of the *j*th inhibitory synapse changes according to the following:

(17) $$\frac{\text{d}w_{j}(t)}{\text{d}t}=\eta_{\text{H}}\left[x_{j}(t)S_{\text{post}}(t)+x_{\text{post}}(t)S_{j}(t)-\alpha_{\text{H}}S_{j}(t)\right],$$

where η_H_ is the learning rate, and α_H_ is a parameter that defines the postsynaptic firing rate. The first two terms on the right-hand side of Equation 17 are Hebbian terms that increase the weights when both presynaptic and postsynaptic activities are correlated. The last term on the right-hand side of Equation 17 is a penalty term for inhibitory spikes alone, which creates a homeostatic setpoint for the postsynaptic firing rate given by the following:

(18) $$\rho_{0}\approx \frac{\alpha_{\text{H}}}{2\tau_{\text{STDP}}}.$$

Later we describe how to arrive at this approximation.

##### Inhibitory synaptic scaling for flat tuning

One of the synaptic weight profiles we used for inhibitory synapses was flat (i.e., all synapse groups had the same mean strength). To learn the flat profile from random initial weights, we implemented a scaling plasticity rule, partially based on experimental work that observed synaptic scaling on inhibitory synapses (Stellwagen and Malenka, 2006; Zhong et al., 2018). Weights are increased if the postsynaptic firing rates are too high, and decreased otherwise, as follows:

$$\frac{\text{d}w_{j}(t)}{\text{d}t}=\eta_{\text{s}}w_{\text{Is}}\left[y_{\text{post}}(t)-\rho_{0}\right]\Theta \left(y_{\text{post}}(t)-\alpha_{\text{s}}\rho_{0}\right)$$

(19) $$-\eta_{\text{s}}w_{j}(t)\left[\rho_{0}-y_{\text{post}}(t)\right]\Theta \left(\frac{\rho_{0}}{\alpha_{\text{s}}}-y_{\text{post}}(t)\right),$$

where η_s_ is a learning rate, *w*_Is_ is a reference weight, ρ_0_ is a firing rate reference value, chosen to be the same as the one for Hebbian plasticity rule, Θ(·) is the Heaviside function, and α_s_ is a term that sets the firing rate range for which synapses do not change. Postsynaptic neuron's firing rate is computed with a slow averaging of the postsynaptic activity through the following:

(20) $$\frac{\text{d}y_{\text{post}}(t)}{\text{d}t}=-\frac{y_{\text{post}}(t)}{\tau_{\text{scaling}}}+\frac{1}{\tau_{\text{scaling}}}S_{\text{post}}(t),$$

where τ_scaling_ is the time constant for the postsynaptic activity and $S_{\text{post}}(t)$ is the postsynaptic spike train (Eq. 5). The last term on the right-hand side of the equation above is divided by τ_scaling_ so that *y*_post_ (*t*) is in units of rate. Synaptic depression is weight-dependent, whereas synaptic potentiation is not, which ensures that all synaptic weights tend to the same value. When the postsynaptic neuron is firing below a threshold $\rho_{0}/\alpha_{\text{s}}$, all inhibitory synapses in the flat group have their weights decreased proportionally to the difference between the target firing rate and the average firing rate, but also proportional to the current weight value. This way, strong synapses undergo stronger decrease than weak ones. Conversely, when the postsynaptic neuron is firing above a threshold α_s_ ρ_0_, all synapses increase in value by the same amount. Intuitively, these mechanisms ensure that all synapses converge to the same value for a long run (see below).

##### Anti-Hebbian inhibitory plasticity

The third inhibitory plasticity rule we used is an anti-Hebbian rule inspired by experimental data (Woodin et al., 2003; Ormond and Woodin, 2009, 2011) and theoretical work on recurrent networks (Hendin et al., 1997). Synaptic weights change according to the following:

(21) $$\frac{\text{d}w_{j}(t)}{\text{d}t}=-\eta_{\text{aH}}(t)\left[x_{j}(t)S_{\text{post}}(t)+x_{\text{post}}(t)S_{j}(t)-\alpha_{\text{aH}}S_{j}(t)\right],$$

where $\eta_{\text{aH}}(t)$ is a variable learning rate and α_aH_ is a parameter to counterbalance the anti-Hebbian term (see also Discussion). The resulting rule dictates that coincident events decrease inhibitory synapses, whereas noncoincident ones increase synaptic weights. Because of the unstable nature of this plasticity rule (see details below), we implemented a time-varying learning rate, which evolves according to the following:

(22) $$\frac{\text{d}\eta_{\text{aH}}(t)}{\text{d}t}=-\frac{\eta_{\text{aH}}(t)}{\tau_{\text{aH}}}+M_{\text{aH}}(t),$$

where τ_aH_ is the decay time constant for the learning rate, and *M*_aH_ (*t*) is an external signal to transiently activate plasticity. We speculate that such signal could come from modulatory neurons, such as dopaminergic or cholinergic and assumed that the external signal peaks at a time *t*_0_ (beginning of the simulation), so that

(23) $$M_{\text{aH}}(t)=\eta_{\text{aH}}^{*}\delta (t-t_{0}),$$

where $\eta_{\text{aH}}^{*}$ is the maximum learning rate before decaying to zero, and *t*_0_ is the time when plasticity at these synapses is initiated. Parameters used for plasticity models are detailed in Table 4.

**Table 4.** Simulation parameters for the plasticity rules

Parameter	Symbol	Value	Figures
STDP time constant	τ_STDP_	20 ms	5, 7, 9
Hebbian learning rate	η_H_	10^−3^	5, 7, 9
Hebbian decay term	α_H_	0.2	5, 7, 9
Firing rate setpoint	ρ_0_	5 Hz	5, 7, 9
Scaling time constant	τ_scaling_	1000 ms	7
Scaling learning rate	η_s_	10^−7^	7
Scaling learning rate weight	*w*_Is_	0.8	7
Scaling threshold parameter	α_s_	2	7
Anti-Hebbian initial learning rate	$\eta_{\text{aH}}^{*}$	10^−3^	9
Anti-Hebbian learning rate time constant	τ_aH_	250 s	9
Anti-Hebbian increase term	α_aH_	0.165	9
Anti-Hebbian peak time	*t*_0_	0 ms	9
Simulation time	—	30 min	5, 7, 9

##### Mean-field analysis of the plasticity rules

We were interested in plasticity rules with stable dynamics. For a better intuition on fixed-point dynamics and stability, we consider here a simplified dynamics of a mean-field model for both the Hebbian (Vogels et al., 2011) and the anti-Hebbian models. We define the postsynaptic firing rate as ν_post_ (*t*) and the presynaptic firing rates as ν*_j_*(*t*). The traces of both presynaptic afferent and postsynaptic neuron thus have an average of $\tau_{\text{STDP}}\nu_{j}(t)$ and $\tau_{\text{STDP}}\nu_{\text{post}}(t)$, respectively (Zenke et al., 2015). Neglecting any correlation between presynaptic and postsynaptic spikes, the average weight change for Hebbian synapses is given by the following:

(24) $$\langle \frac{\text{d}w_{j}(t)}{\text{d}t}\rangle =\eta_{\text{H}}\left[2\tau_{\text{STDP}}\nu_{j}(t)\nu_{\text{post}}(t)-\alpha_{\text{H}}\nu_{j}(t)\right],$$

where 〈·〉 represents average over time. Intuitively, the postsynaptic firing rate, ν_post_ (*t*), changes negatively with changes in inhibitory weights: increased inhibition generates fewer postsynaptic spikes and vice versa for decreased inhibition. This means that average firing rates are inversely linked to average inhibitory weights, as follows:

(25) $$〈\frac{\text{d}\nu_{\text{post}}(t)}{\text{d}t}〉\propto -〈\frac{\text{d}w_{j}(t)}{\text{d}t}〉=2\eta_{\text{H}}\nu_{j}(t)\tau_{\text{STDP}}\left[\frac{\alpha_{\text{H}}}{2\tau_{\text{STDP}}}-\nu_{\text{post}}(t)\right].$$

The steady state is computed by considering the vanishing point of the equation above (we assume that the presynaptic activity is nonzero); thus,

(26) $$\nu_{\text{post}}(t)=\frac{\alpha_{\text{H}}}{2\tau_{\text{STDP}}}\equiv \rho_{0}.$$

This means that the postsynaptic activity ν_post_(*t*) increases (via reduction in inhibitory efficacy) when below ρ_0_ and decreases when above ρ_0_, creating a stable fixed point for the postsynaptic firing rate.

The opposite is true for the anti-Hebbian plasticity rule. Changes in postsynaptic firing rate (with the same assumption as for the Hebbian plasticity rule) follow the following:

(27) $$〈\frac{\text{d}\nu_{\text{post}}(t)}{\text{d}t}〉\propto \nu_{\text{post}}(t)-\frac{\alpha_{\text{aH}}}{2\tau_{\text{STDP}}}=\nu_{\text{post}}(t)-\rho_{1}.$$

Because postsynaptic activity increases when it is above threshold ρ_1_ and decreases when it is below, this rule is unstable. The postsynaptic firing rate eventually explodes or vanishes. We chose the simplest way to overcome these problems by setting a time-varying learning rate. Other intricate mechanisms could be implemented, but this is not the scope of our work.

##### Convergence of weights following the scaling plasticity rule

Our scaling plasticity rule has two different mechanisms: one for LTD and one for LTP. LTD is multiplicative, and LTP is additive (Eq. 19). The combined effect ensures that all incoming weights collapse to the same value (synaptic changes do not depend on presynaptic activity either). Here we present a mathematical intuition to explain how synaptic weights can converge to the same value. First, we rewrite the scaling plasticity rule into two simplified terms. We consider constant postsynaptic firing rate during LTD, $y_{\text{post}}(t)=\rho_{0}-{\bar{y}}_{\text{post}}^{\text{LTD}}$, with $y_{\text{post}}(t)<\rho_{0}/\alpha_{\text{s}}$. Consequently, the LTD part is described by the following:

(28) $$\frac{\text{d}w_{j}(t)}{\text{d}t}=-\eta_{\text{s}}w_{j}(t){\bar{y}}_{\text{post}}^{\text{LTD}}$$

with solution as follows:

(29) $$w_{j}(t)=w_{j}(0)\exp\left(-\eta_{\text{S}}{\bar{y}}_{\text{post}}^{\text{LTD}}t\right).$$

Doing the same for LTP ($y_{\text{post}}(t)={\bar{y}}_{\text{post}}^{\text{LTP}}+\rho_{0}$, with $y_{\text{post}}(t)>\rho_{0}\alpha_{\text{s}}$), we arrive at the following:

(30) $$\frac{\text{d}w_{j}(t)}{\text{d}t}=\eta_{\text{s}}w_{\text{Is}}{\bar{y}}_{\text{post}}^{\text{LTP}}$$

with solution as follows:

(31) $$w_{j}(t)=w_{j}(0)+\eta_{\text{s}}w_{\text{Is}}{\bar{y}}_{\text{post}}^{\text{LTP}}t.$$

Defining $t_{i}^{\text{LTD}}$ as the *i*th interval in which LTD occurred and $t_{i}^{\text{LTP}}$ as the *i*th interval in which the synapse underwent LTP, we can combine Equations 29 and 31 to rewrite the synaptic strength, *w_j_*(*t*), at time *t* as follows:

$$w_{j}(t)=w_{j}(0)\exp\left(-\eta_{\text{s}}{\bar{y}}_{\text{post}}^{\text{LTD}}\sum_{i=1}^{T_{\text{LTD}}}t_{i}^{\text{LTD}}\right)$$

(32) +ηswIsy¯postLTP∑i=1TLTPtiLTP exp(−ηsy¯postLTD∑k=i+1TLTDtkLTD),

where *T*_LTD_ and *T*_LTP_ are the number of intervals with LTD and LTP, respectively. We assume that LTP is always followed by LTD in Equation 32 for simplicity, and thus *T*_LTD_ =*T*_LTP_ ± 1. The first term on the right-hand side of Equation 32 vanishes for long times, and the second term dominates with the late terms (*i* >> 1, or, from $T_{\text{LTP}}-\kappa$ to *T*_LTP_ for small κ), as follows:

(33) limt→∞wj(t)≈ηswIsy¯postLTP∑i=TLTP−κTLTPtiLTP exp(−ηsy¯postLTD∑k=i+1TLTDtkLTD),

which is finite given that the postsynaptic neuron's firing rate fluctuates around the target firing rate, ρ_0_, and does not depend on the initial weight *w_j_*(0).

#### Correlation

We quantified the response of the postsynaptic neuron to natural inputs with the Pearson correlation between postsynaptic firing rate and input firing rate fluctuations, per signal group. We computed the firing rate of a signal groups as the low-pass filter of the spike trains of its excitatory afferents, as follows:

(34) $$\tau_{Z}\frac{\text{d}Z_{\mu }(t)}{\text{d}t}=-Z_{\mu }(t)+\sum_{j\subset \mu }S_{j}(t),$$

where *Z*_μ_(*t*) is the firing rate of the signal group μ at time *t*, filtered with a time constant τ*_Z_*. The postsynaptic activity is also computed through a low-pass filter of its spike train, as follows:

(35) $$\tau_{Y}\frac{\text{d}Y(t)}{\text{d}t}=-Y(t)+S_{\text{post}}(t),$$

where *Y*(*t*) is the activity of the postsynaptic neuron at time *t* filtered with a time constant τ*_Y_*. The correlation is then computed as follows:

(36) $$C_{\mu }=\frac{cov(Z_{\mu },Y)}{\sigma_{Z_{\mu }}\sigma_{Y}}=\frac{〈(Z_{\mu }-〈Z_{\mu }〉)(Y-〈Y〉)〉}{\sqrt{〈{\left(Z_{\mu }-〈Z_{\mu }〉\right)}^{2}〉〈{\left(Y-〈Y〉\right)}^{2}〉}},$$

where *cov*(*z*, *y*) is the covariance between variables *z* and *y*, σ*_z_* is the SD of variable *z*, and 〈·〉 represents time average.

Subsequently, we computed a performance index Δ*C* as the difference between the correlation measure for preferred (μ = 9) and nonpreferred (μ = 1) input signals, as follows:

(37) $$\Delta C=\frac{1}{2}\left(C_{9}-C_{1}\right).$$

Maximum positive performance index (Δ*C* = 1) means that the preferred signal group has maximum correlation (*C*_9_ = 1) while the nonpreferred signal group has maximum anticorrelation (*C*_1_ = −1), indicating that the postsynaptic neuron is responding solely to the preferred signal group. Consequently, Δ*C* = −1 indicates that the postsynaptic neuron is responding solely to the nonpreferred signal group. A flat response is indicated by Δ*C* = 0. Maximum Δ*C* (either positive or negative) is only achievable if the preferred (signal group 9) and nonpreferred (signal group 1) are strictly anticorrelated. This is unlikely given that all input signals are independent, and they will feature periods of coactivation. We define as best performance when Δ*C* = 0 for all inhibitory inputs active (control), Δ*C* = 1 (or Δ*C* > 0) for one inhibitory population inactive, and Δ*C* = –1 (or Δ*C* < 0) when the other inhibitory population is inactive. Parameters used for computing correlations are detailed in Table 5.

**Table 5.** Simulation parameters for the correlation measure

Parameter	Symbol	Value	Figures
Presynaptic time constant	τ_Z_	10 ms	4, 6, 8, 10, 11
Postsynaptic time constant	τ_Y_	250 ms	4, 6, 8, 10, 11
Simulation time	—	30 min	4, 6, 8, 10, 11

The SD of the output firing rate (see Figs. 4*C*, 8*C*, 10*C*) was computed with a 1 s bin. The coefficient of variation of the interspike interval (CV_ISI;_ see Figs. 4*C*, 8*C*, 10*C*) was calculated as the SD of the interspike interval (ISI) of the output spike train divided by the mean ISI.

#### Software and code availability

Simulations were run in Fortran, compiled with Intel Fortran Compiler 19.0 on an Intel-based Linux computer (Debian 9; i9-9900× processor; 32 GB memory). Codes are available online (https://github.com/ejagnes/flexible_switch_2ISP/). Individual plots were generated with Gnuplot (http://www.gnuplot.info/). Figures were generated with Inkscape (https://inkscape.org/).

## Results

**Figure 2.**
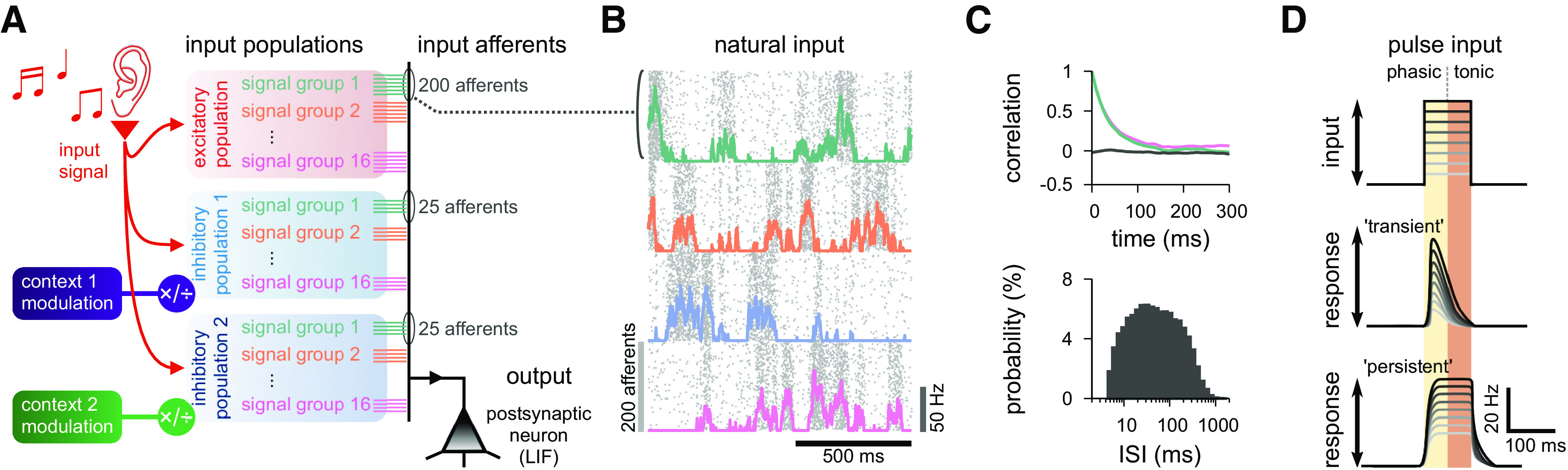
Model details. ***A***, Schematic of the input organization. An external signal (representing, e.g., sound) was delivered through three input populations (one excitatory and two inhibitory), with 16 input signals per population (representing, e.g., sound frequency). Each signal was simulated by 250 independent, but temporally correlated, spike trains (input afferents); 200 excitatory, and 50 inhibitory divided into two groups of 25. One postsynaptic neuron (black triangle) was the output of this system, simulated as a single-compartment LIF neuron. The firing rate of each of the inhibitory populations was modulated by a contextual cue (green and purple boxes). Excitatory and inhibitory input spike trains were generated as point processes (for details, see Materials and Methods). ***B***, Natural input statistics. Raster plot (gray dots) of 800 neurons that take part in 4 signal groups (200 neurons per signal group), each with firing rate changing according to a modified OU process (colored lines; see Materials and Methods). ***C***, Temporal autocorrelation (top) and distribution of the ISIs (bottom) of the presynaptic inputs. The autocorrelation of two groups are shown (green and pink), as well as the correlation between two different groups (black). Autocorrelation is computed as the Pearson coefficient with a delay (*x* axis). ***D***, Pulse input schematic. A steplike increase in the firing rate of a given input group lasting 100 ms (top) with varying firing rates (grayscale). The postsynaptic response can be separated in phasic (first 50 ms), and tonic (last 50 ms), which reveals transient (middle) or persistent (bottom) types of response.

To study the effect of interacting populations of feedforward inhibition, we investigated the response of a single postsynaptic LIF neuron receiving tuned excitatory inputs and inhibition from two distinct populations. Excitatory inputs were organized into a single population, subdivided into 16 signal groups of 200 excitatory afferents. Inhibitory inputs initially formed a single population, mirroring the excitatory subdivision, but with 50 afferents per group. Subsequently, we split the inhibitory inputs into two populations with 25 afferents per signal group (Fig. 2*A*; see Materials and Methods), allowing us to obtain two differently tuned populations (presumably types) of inhibition. Excitatory and inhibitory afferents belonging to the same group shared temporal fluctuations in firing rates, termed input patterns, even if they belonged to different populations. In our simulations, input patterns could either be natural or pulse. Natural inputs were generated through an inhomogeneous Poisson process based on a modified OU process (Fig. 2*B*,*C*), such that neurons of the same signal group also had temporally correlated firing patterns (Fig. 2*C*, top) (see Ujfalussy et al., 2018). The resulting long-tail distribution of ISIs (Fig. 2*C*, bottom) was similar to experimentally observed spike patterns *in vivo* (Reich et al., 2000; Hengen et al., 2013). We used this type of input to train inhibitory synapses via plasticity rules, and to quantify steady-state (average) postsynaptic responses.

In the alternative pulse input regimen, we analyzed transient responses with 100-ms-long pulses of varying amplitudes (Vogels et al., 2011). Pulses were delivered through a single signal group of excitatory and inhibitory afferents, whereas all other groups remained at baseline firing rate (see Materials and Methods). Responses were quantified according to postsynaptic firing rates during the first (phasic) and last (tonic) 50 ms stimulation (Fig. 2*D*), averaged over 100 trials. Separating responses in phasic and tonic allowed us to discriminate changes in output because of the input onset (Fig. 2*D*, transient), when excitation outweighs inhibition (phasic period), and slower integration of the pulse (Fig. 2*D*, persistent), when excitation and inhibition are balanced (tonic period). The transient period is best captured by limiting the phasic window to 50 ms, and we thus set the tonic window to the same duration to keep both window sizes symmetric.

**Figure 3.**
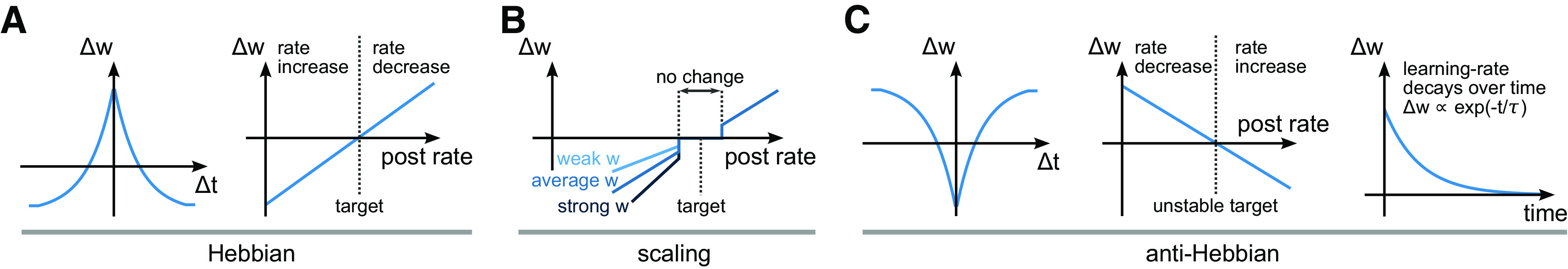
Synaptic plasticity models. ***A***, Hebbian plasticity rule. Left, Spike-timing dependency. Δ*w* indicates level of synaptic change, and Δ*t* indicates interval between presynaptic and postsynaptic spikes. Coincident presynaptic and postsynaptic spikes elicit positive changes, whereas presynaptic spikes alone elicit negative changes in synaptic strength (Vogels et al., 2011). Right, Synaptic changes (Δ*w*) as a function of postsynaptic firing rate. When the postsynaptic neuron's firing rate is above the target rate, inhibitory synapses increase in weight and, as a consequence, the postsynaptic neuron's firing rate decreases. The opposite happens for when the postsynaptic neuron's firing rate is lower than the target rate (Vogels et al., 2011) (see Materials and Methods). ***B***, Synaptic scaling rule. Changes in synaptic strength (Δ*w*) as a function of the postsynaptic neuron's firing rate. When the postsynaptic neuron's firing rate is lower than a lower bound threshold, inhibitory synapses decrease, proportionally to their current strength. When the postsynaptic neuron's firing rate is higher than an upper bound threshold, inhibitory synapses increase. Because of the lower and upper bounds, there is a region with no change around the target rate. ***C***, Anti-Hebbian plasticity rule. Left, Spike-timing dependency. Presynaptic spikes elicit positive changes, whereas coincident presynaptic and postsynaptic spikes elicit negative changes in synaptic weights. Middle, Changes in synaptic efficacy (Δ*w*) as a function of the postsynaptic firing rate. The target rate of anti-Hebbian plasticity rule is unstable. Right, Evolution of the learning rate of the anti-Hebbian plasticity model. Because of its unstable nature, we set the learning rate to decay exponentially over time.

Learning was implemented via three distinct inhibitory plasticity rules (Fig. 3), in three different combinations. We first implemented a Hebbian rule, which potentiated synaptic weights for coincident presynaptic and postsynaptic spikes and depressed them for sole presynaptic spikes (Vogels et al., 2011) (Fig. 3*A*), in one of the two inhibitory populations, while the synapses of the other inhibitory and the excitatory population remained fixed. This learning rule has previously been shown to generate inhibitory weight profiles that mirror the excitatory synaptic weight profiles of a postsynaptic neuron, imposing a firing rate fixed point (target; Fig. 3*A*) by balancing excitation and inhibition (Vogels et al., 2011), supporting similar experimental findings in mouse auditory cortex (D'amour and Froemke, 2015). Next, we implemented the Hebbian plasticity rule in one of them and a scaling plasticity rule (Fig. 3*B*) in the other population. The homeostatic scaling rule upregulates or downregulates the entire synapse population to reach a predetermined target firing rate. Notably, this plasticity rule was purely local, taking only synaptic weights and postsynaptic firing rate into account, similarly to the experimentally observed scaling of inhibitory synapses (Stellwagen and Malenka, 2006; Zhong et al., 2018). Finally, we also implemented an experimentally inspired (Woodin et al., 2003; Ormond and Woodin, 2009, 2011) anti-Hebbian rule in the second inhibitory population (Fig. 3*C*). Unlike its Hebbian counterpart, the anti-Hebbian rule leads to indefinite increases in the firing rate of the postsynaptic neuron because correlated activity decreases synaptic weights (only sole presynaptic spikes increase synaptic weights; see Materials and Methods). The anti-Hebbian plasticity rule is thus unstable (Fig. 3*C*, middle). We found that we could prevent catastrophe without incorporating additional, complex dynamics by using a variable learning rate for the anti-Hebbian rule. For simplicity, we decreased the learning rate exponentially over time (Fig. 3*C*, right), but this could also be achieved through top-down control (see Discussion).

### Shaping and modulating a single inhibitory population

**Figure 4.**
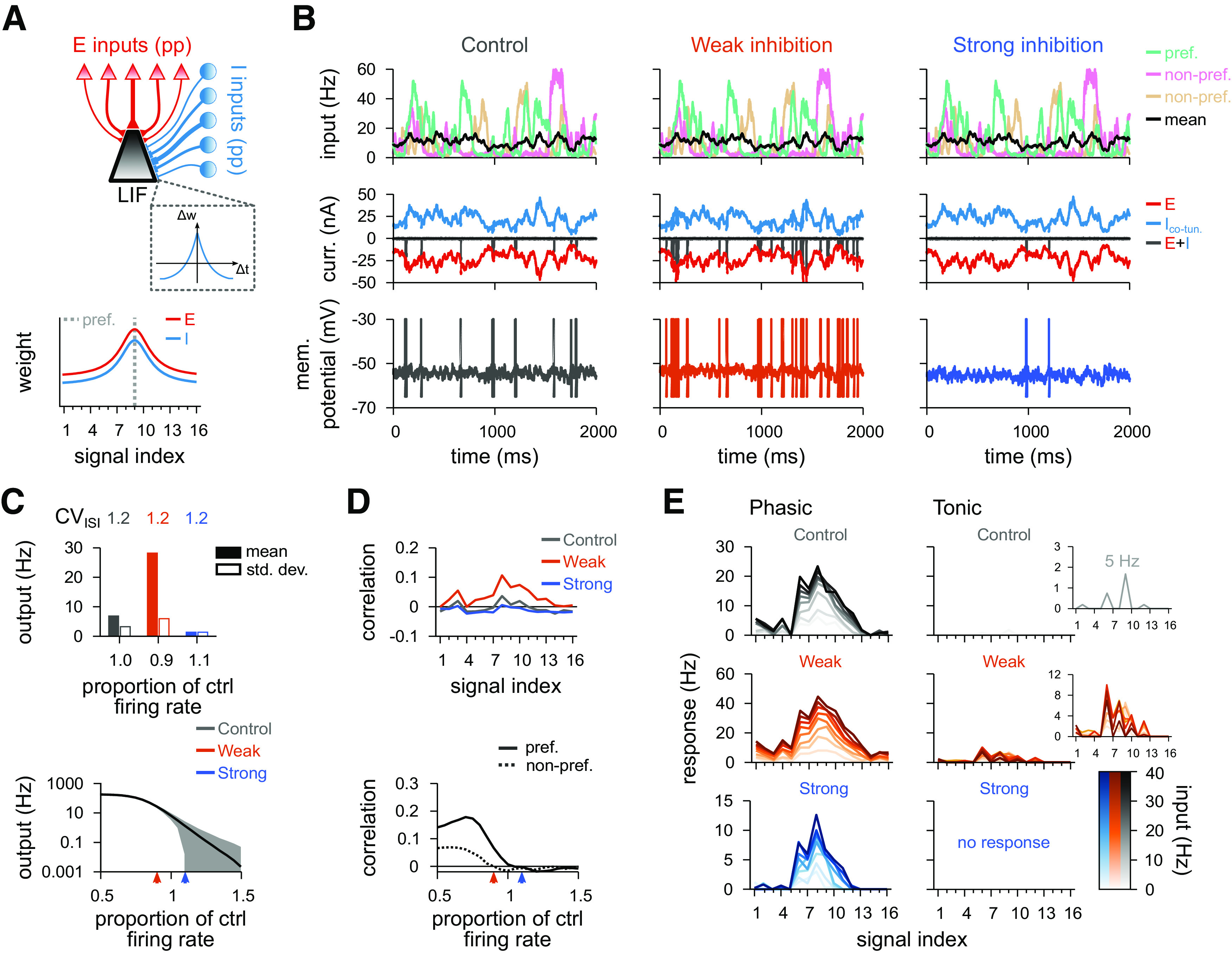
Postsynaptic response for a model with a single inhibitory population. ***A***, Schematic of the circuit with a single inhibitory population (top). Presynaptic spikes were generated as point processes (pp), for both excitatory (red; 16 signals) and inhibitory (blue; 16 signals) inputs, and fed into a single-compartment LIF neuron. Schematic of the synaptic weight profiles (bottom). Average weight (*y* axis) for different input signals (*x* axis); preferred signal is pathway no. 9 (gray dashed line). ***B***, Firing rate of the preferred, and two nonpreferred inputs and mean of all inputs (top row), excitatory and inhibitory input currents (middle row), and membrane potentials (bottom row), for control (left), decreased (middle), and increased (right) inhibition. Decreased (increased) inhibition lowered (raised) inhibitory firing rates by 10%, respectively. ***C***, CV_ISI_, and mean and SD of the postsynaptic firing rate in response to natural input for the 3 explored cases (top), and as a function of the inhibitory firing rate (bottom). Arrowheads indicate the analyzed cases. ***D***, Pearson correlation between postsynaptic firing rate and excitatory input firing rates for different input signals for the three conditions in ***B*** (top). Correlation between output activity and preferred (continuous line) or nonpreferred (dashed line) inputs as a function of the inhibitory firing rate (bottom). ***E***, Response to a pulse input in the phasic (left; first 50 ms) and tonic (right; last 50 ms) periods. Firing rate computed as the average number of spikes (for 100 trials) normalized by the bin size (50 ms). Each line corresponds to a different input strength: from light (low-amplitude pulse) to dark (high-amplitude pulse) colors. Insets, Tonic response for control and decreased inhibitory firing rates.

To begin, we constructed a standard cortical circuit motif with one excitatory and one inhibitory population (Diesmann et al., 1999; Vogels and Abbott, 2009; Vogels et al., 2011; Clopath et al., 2016; Weber and Sprekeler, 2018) (Fig. 4*A*, top). We followed previous work showing that the Hebbian plasticity rule (Fig. 3*A*) changes inhibitory synapses to provide precisely balanced inputs (Vogels et al., 2011), such that both excitatory and inhibitory weight profiles are shaped according to previous experimental observations (Froemke et al., 2007) (Fig. 4*A*, bottom). Afferent synaptic weights of the excitatory population were set according to a receptive field tuning curve (see Materials and Methods) while the efficacy of inhibitory afferents was governed by a synaptic plasticity rule for a period of 30 min. Learning was established by a Hebbian plasticity rule that potentiated synapses for coincident presynaptic and postsynaptic spikes and depressed synapses for sole presynaptic spikes (Vogels et al., 2011) (Fig. 3*A*). The plasticity rule was set to allow average postsynaptic firing rates of ∼5 Hz for natural inputs (Fig. 4*B*,*C*). We then tested the effect of changing the gain of all inhibitory afferents, while keeping their synaptic strengths fixed, by modulating their firing rates, from 50% to 150% of control rates. This change of input balance translated into changes in output rates (Fig. 4*C*, bottom) and spike patterns (Fig. 4*B*, middle and right). When inhibition was equal or larger than excitation, the output was largely uncorrelated to any given input signal (Fig. 4*D*, top). When inhibitory firing rates fell below 90% of the control condition, the output first began to correlate with the preferred input signal. When inhibition became even weaker, the correlations increased, and even nonpreferred signals were articulated in the postsynaptic firing patterns (Fig. 4*D*, bottom).

Transient presynaptic activity pulses caused strong phasic responses in the balance state when they were delivered through the afferents of the preferred inputs (Fig. 4*E*, top row). Stimuli from nonpreferred afferents were largely ignored. This discriminability between transients of low- or high-amplitude pulses decreased when inhibition was downregulated (Fig. 4*E*, middle row) such that pulse stimuli from all signal groups caused a response. Increased inhibition, on the other hand, completely abolished transient responses to nonpreferred afferents (Fig. 4*E*, bottom row). In all 3 cases (balanced control, weak and strong inhibition), the postsynaptic neuron elicited most of its spikes within the phasic period of the total 100 ms input step (Fig. 4*E*). This indicates that strong postsynaptic responses are mostly driven by the onset of the presynaptic stimulation rather than the stimulus being integrated slowly over time, a consequence of the precise balance of excitatory and inhibitory inputs (Vogels et al., 2011).

Thus, a single inhibitory population, even with tuned weights, could not affect the postsynaptic receptive field via only the modulation of the inhibitory firing rate. To test whether an additional inhibitory population would allow for more sophisticated control of postsynaptic activity, we constructed a model with different plasticity rules, which were applied to two different populations of inhibitory inputs.

### Plasticity shapes inhibitory weight profiles and receptive fields

**Figure 5.**
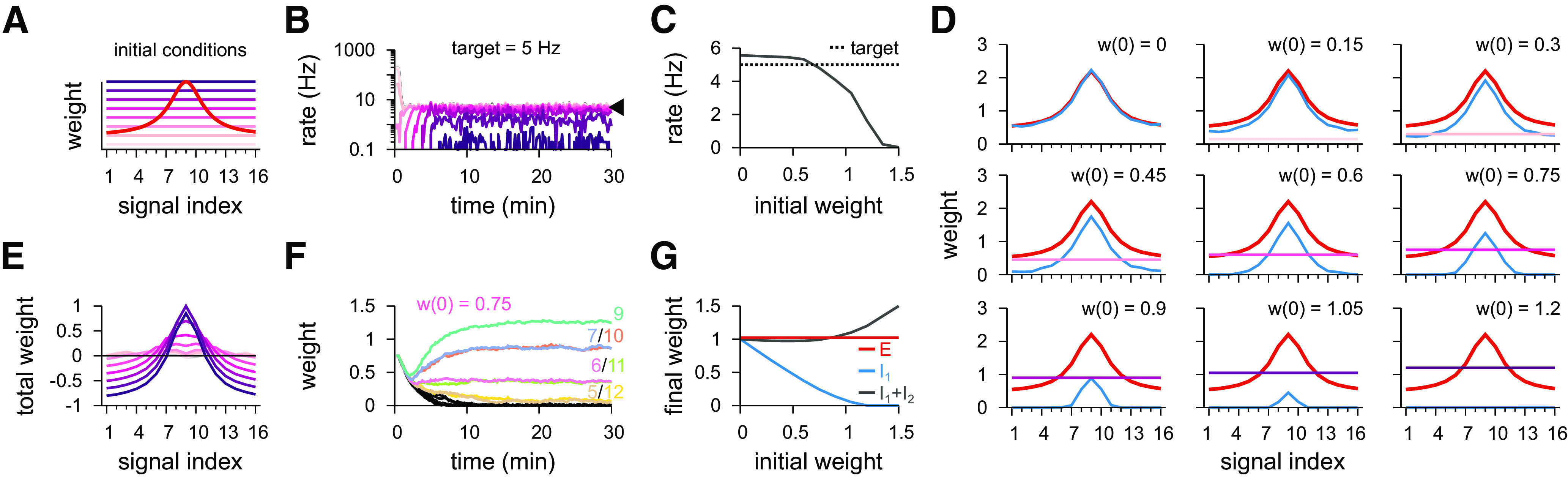
Inhibitory plasticity acting on one inhibitory population compensates global inhibition from a second inhibitory population. ***A***, Schematic of the synaptic weight profile for excitatory synapses (red) and different initial conditions for inhibitory synapses (pink to purple color code). Inhibitory Population 1 has its inhibitory synapses changing according to a plasticity mechanism, whereas Population 2 remains fixed. ***B***, Time course of the postsynaptic firing rate for different initial conditions (colors as in ***A***). Inhibitory plasticity on Population 1 is set to achieve a balanced state with target of 5 Hz (arrowhead). ***C***, Stabilized postsynaptic firing rate as a function of the initial inhibitory synaptic weight. ***D***, Individual synaptic weight profiles for excitatory (red), inhibitory Population 1 (blue, after synaptic stabilization), and inhibitory Population 2 (colors as in ***A***). ***E***, Total synaptic weight per signal (excitatory – inhibitory) for different initial conditions after stabilization of synapses from Population 1. ***F***, Example of synaptic dynamics of inhibitory Population 1 for a given initial condition. Colors represent different signal groups. ***G***, Final weights as a function of initial inhibitory weights. Plotted are excitatory (red), plastic inhibitory (blue), and sum of total inhibitory synapses (gray).

To study how plasticity can shape the emergence of distinct synaptic weight profiles, we incorporated inhibitory synaptic plasticity mechanisms into a model with two inhibitory populations. We started with a symmetric Hebbian plasticity rule in one of the two inhibitory populations: coincident presynaptic and postsynaptic spikes potentiated synapses, whereas sole presynaptic spikes depressed synapses (Vogels et al., 2011) (Fig. 3*A*). The synapses of the excitatory and the other inhibitory population remained fixed (Fig. 5). Simulations began with tuned excitatory synapses and flat inhibitory weight profiles in both inhibitory populations (Fig. 5*A*).

**Figure 6.**
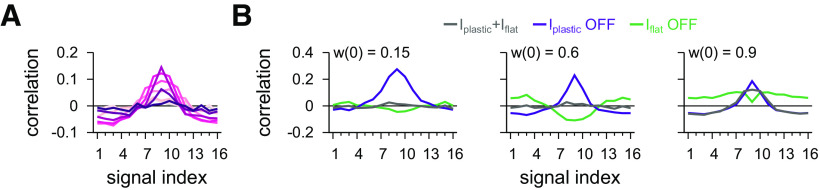
Postsynaptic response after stabilization of synapses from one population. ***A***, Pearson correlation between postsynaptic firing rate and excitatory input firing rates with both inhibitory populations active. Color code as in Figure 5*A*. ***B***, Pearson correlation between postsynaptic firing rate and excitatory input firing rates for different signals with both inhibitory populations active, Population 1 inactive, and Population 2 inactive. Three examples are shown.

After 30 min of stimulation with natural inputs (compare Fig. 2*B*), inhibitory weights of the plastic population stabilized (Fig. 5*D–G*). Whether the target firing rate (Fig. 5*B*,*C*) was reached depended on the synaptic strength of the other, static population of inhibitory synapses. If the static weights were weak, the plastic synapses increased their strength until the target firing rate was reached (Fig. 5*C*). If the static population provided strong inhibition (and thus kept postsynaptic firing below the target rate), weights from the plastic population would eventually vanish, before the target firing rate could be reached (Fig. 5*C*,*G*). Consequently, the shape of the static population determined the shape of the plastic population (Fig. 5*D*,*E*). As expected, the input/output correlation of the postsynaptic responses followed the effective synaptic weight profile (Fig. 6*A*, compare Fig. 5*E*), with distinct input/output correlations for turning either of the populations off (Fig. 6*B*). The Hebbian plasticity rule, because of the strengthening of synapses for coincident presynaptic and postsynaptic spikes, thus complemented additional inhibitory synaptic connectivity in establishing a state of detailed balance of excitatory and inhibitory inputs.

### Hebbian and scaling plasticity rules

**Figure 7.**
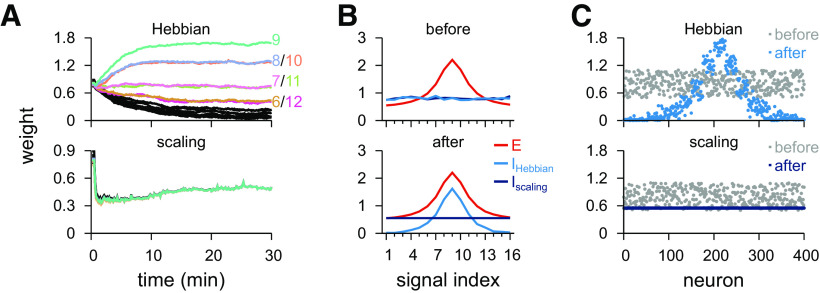
Simultaneous learning of two inhibitory profiles via Hebbian and homeostatic scaling plasticity rules. ***A***, Temporal evolution of inhibitory synaptic weights when one inhibitory population follows a Hebbian plasticity rule (top), and the other population follows a synaptic scaling plasticity rule (bottom). ***B***, Initial (top) and final (bottom) weight profiles from ***A***, with excitatory weights for reference. ***C***, Individual synaptic weights before and after learning for synapses following the Hebbian plasticity rule (top) and synapses following the scaling plasticity rule (bottom).

Next, we introduced plasticity to the second population of inhibitory afferents. We tested two different rules, beginning with a homeostatic plasticity rule, which (multiplicatively) scaled synapses down and additively potentiated synapses so that a fixed point for the postsynaptic firing rates was reached (Fig. 3*B*; see Materials and Methods). With the homeostatic rule coactive, the Hebbian synapses, connections changing according to the Hebbian plasticity rule, developed a co-tuned profile from initially random weights (Fig. 7*A*, top; Fig. 7*C*, left), while the synapses following the scaling rule collapsed to a single value (Fig. 7*A*, bottom; Fig. 7*C*, right; for mathematical analysis, see Materials and Methods). Consequently, the postsynaptic neuron received precisely balanced inputs (Fig. 7*B*). The two plasticity rules cooperate to impose an average postsynaptic activity, and thus naturally work in harmony. Importantly, both plasticity rules are stable regarding their interaction with postsynaptic dynamics (see Materials and Methods), and as such, not sensitive to initial conditions, and relatively robust to parameter choices, as long as the postsynaptic activity fixed point (see Materials and Methods) imposed by both plasticity rules match.

**Figure 8.**
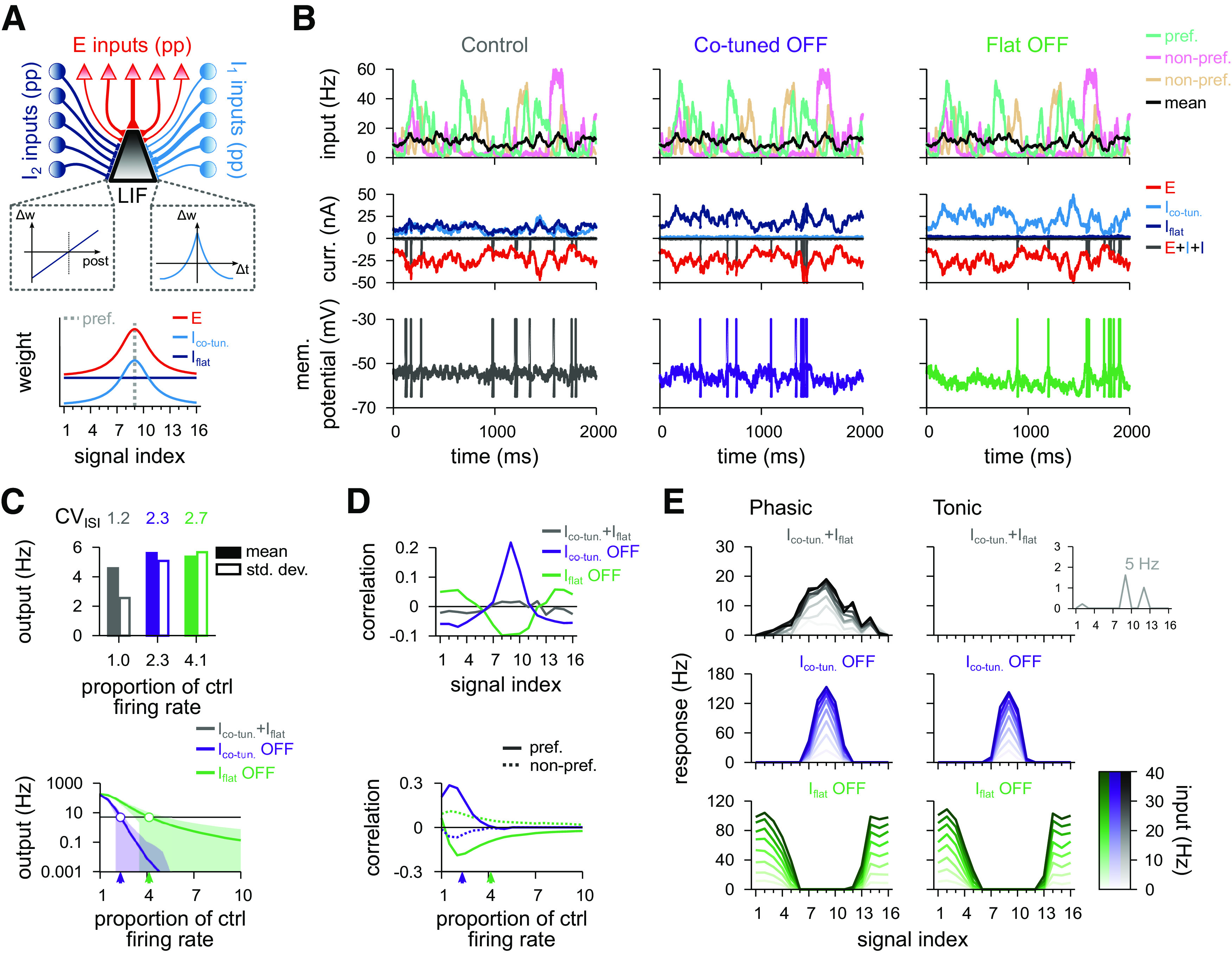
Postsynaptic response for the model with co-tuned and flat inhibitory populations. ***A***, Schematic of the circuit with two inhibitory populations (top): *I*_1_, co-tuned population; *I*_2_, flat population. Presynaptic spikes were generated as point processes (pp) and fed into an LIF. Schematic of the synaptic weight profile (bottom). Average weight (*y* axis) for different input signals (*x* axis); preferred signal is pathway no. 9 (gray dashed line). ***B***, Firing rate of the preferred and two nonpreferred inputs and mean of all inputs (top row), total excitatory current and inhibitory currents of both populations (middle row), and membrane potential (bottom row), for control (left), co-tuned (middle), and flat (right) population inactive. ***C***, CV_ISI_ and postsynaptic firing rate (mean and SD) in response to natural input for the 3 cases (top). Output firing rate as a function of the firing rate of the (compensatory) active inhibitory population (bottom). Arrowheads indicate the analyzed cases where output rate is equal to 5 Hz (i.e., where the green and purple lines cross the black line). ***D***, Pearson correlation between postsynaptic firing rate and excitatory input firing rates for different input signals for the three conditions in ***B***. Correlation between output activity and preferred (continuous line) or nonpreferred (dashed line) as a function of the inhibitory firing rate of each inhibitory population (bottom). ***E***, Response to a pulse input in the phasic (left; first 50 ms) and tonic (right; last 50 ms) periods. Firing rate computed as the average number of spikes (for 100 trials) normalized by bin size (50 ms). Each line corresponds to a different input strength: from light (low-amplitude pulse) to dark (high-amplitude pulse) colors. Insets, Tonic response for control firing rates.

We then studied the effects of differentially modulating the activity of the two inhibitory populations after their tuning curves had been established by the plasticity rules described above. First, we focused on the interaction of the connectivity created by Hebbian and the scaling plasticity rules (Fig. 8*A*, top; compare Fig. 7), that is, a co-tuned population and a flat population (Fig. 8*A*, bottom). We compared the output of the neuron in three scenarios: with both inhibitory populations active (control); with the co-tuned population inactive; and with the flat population inactive (Fig. 8*B–E*).

With both populations active, the input/output correlation was indistinguishable from a model with one, homogeneous inhibitory population (Fig. 8*D*, top; compare Fig. 4*D*, top), because the two populations (co-tuned and flat) created the same effect as the single (co-tuned) population. Deactivating either population had pronounced effects on postsynaptic responses. We increased the firing rate of the active inhibitory population to maintain the same average output firing rate of 5 Hz in the modulated conditions (Fig. 8*C*, top; where the green and purple lines cross the black horizontal line in Fig. 8*C*, bottom). Fluctuations in firing rate and membrane potential increased in both cases (Fig. 8*B*,*C*). When the co-tuned inhibitory population was turned off, the emerging imbalance of excitation and inhibition unmasked the excitatory tuning curve, thus increasing the chance of action potential generation when preferred signal populations were active (Fig. 8*B*, middle). The compensatory increase in the activity of the flat population further quenched nonpreferred excitatory signals, leading to anticorrelated responses for nonpreferred input signals (Fig. 8*D*, purple), reflecting the lack of postsynaptic firing during periods in which nonpreferred signals were active (Fig. 8*B*, middle). The opposite effect could be observed when the flat population was deactivated. In this case, the lack of inhibition for nonpreferred signals gave rise to input/output correlations for nonpreferred signals, while preferred signals saw no response (Fig. 8*B*, right; Fig. 8*D*, green).

Transient responses, when compared with the unmodulated control case (Fig. 8*E*, top), were substantially increased for preferred inputs when the co-tuned population was deactivated, and the response to nonpreferred signals was completely diminished (Fig. 8*E*, middle). When the flat population was deactivated, the postsynaptic neuron responded strongly to the nonpreferred inputs, but not to preferred inputs (Fig. 8*E*, bottom). Interestingly, modulating either of the inhibitory populations had similar effects on the postsynaptic response both in phasic and tonic periods, in contrast with the unmodulated control case, in which only phasic responses were postsynaptically elicited (Fig. 8*E*). Again, this reflects the state of balance between excitation and inhibition in the unmodulated control case, which only reveals transient input dynamics.

### Hebbian and anti-Hebbian plasticity rules

**Figure 9.**
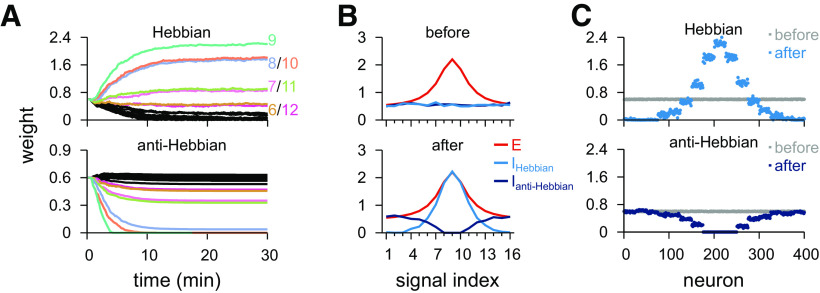
Simultaneous learning of two inhibitory profiles via Hebbian and anti-Hebbian plasticity rules. ***A***, Temporal evolution of inhibitory synaptic weights when one inhibitory population follows a Hebbian plasticity rule (top), and the other population follows an anti-Hebbian plasticity rule (bottom). ***B***, Initial (top) and final (bottom) weight profiles from ***A***, with excitatory weights for reference. ***C***, Individual synaptic weights before and after learning for synapses following the Hebbian plasticity rule (top) and synapses following the anti-Hebbian plasticity rule (bottom).

Instead of a purely homeostatic scaling rule, we also tried an experimentally inspired (Woodin et al., 2003; Ormond and Woodin, 2009, 2011) anti-Hebbian rule in the second inhibitory population (Fig. 3*C*). The anti-Hebbian rule, unlike the Hebbian, decreases synaptic weights for correlated activity, and sole presynaptic spikes increase synaptic weights. Such a rule can only either indefinitely increase the firing rate of the postsynaptic neuron or decrease it to zero (see Materials and Methods). We accounted for the unstable nature of the anti-Hebbian plasticity rule (Fig. 3*C*, middle) by controlling its learning rate, such that it decreased exponentially over time (Fig. 3*C*, right). With both Hebbian and anti-Hebbian rules active, initially random weights evolved into co-tuned and counter-tuned synaptic weight profiles (Fig. 9*A*). As learning slowed down because of the decreasing learning rate, the anti-Hebbian synapses (connections changing according to the anti-Hebbian plasticity rule) stabilized, and Hebbian synapses ceased to change once the target firing rate was reached (Fig. 9*B*,*C*). The anti-Hebbian plasticity rule, differently from the Hebbian one, is sensitive to initial conditions and choice of parameters because of its intrinsic instability (see Materials and Methods). For example, different initialization of inhibitory weights requires distinct decay times for the learning rate (data not shown). Additional complexity in the formulation of the plasticity rule may solve these instability problems (Discussion).

**Figure 10.**
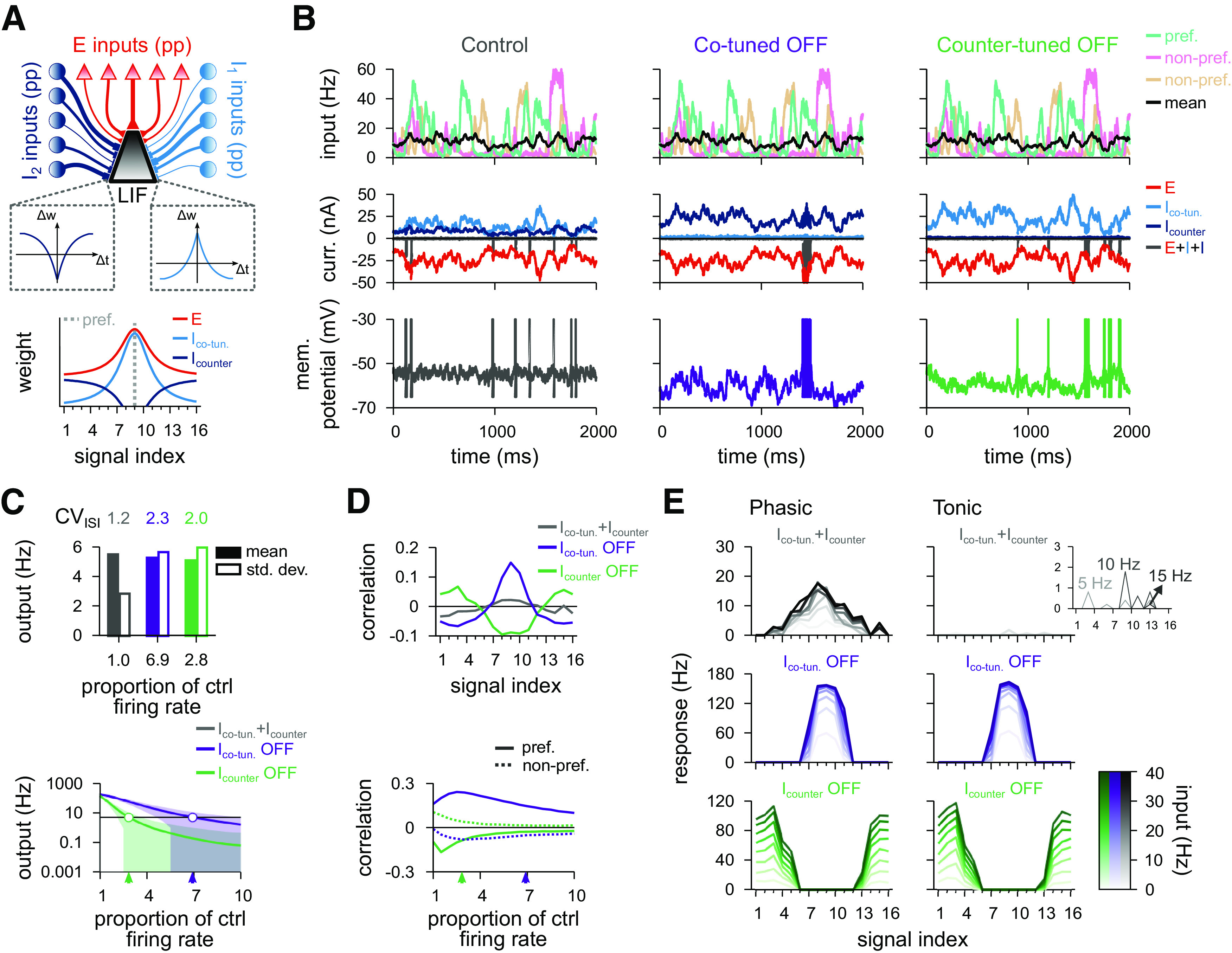
Postsynaptic response for the model with co- and counter-tuned inhibitory populations. ***A***, Schematic of the circuit with two inhibitory populations (top): *I*_1_, co-tuned; *I*_2_, counter-tuned population. Presynaptic spikes were generated as point processes (pp) and fed into an LIF. Synaptic weight profile (bottom). Average weight (*y* axis) for different input signals (*x* axis); preferred signal is pathway no. 9 (gray dashed line). ***B***, Firing rate of the preferred and two nonpreferred inputs and mean of all inputs (top row), total excitatory current and inhibitory currents of both populations (middle row), and membrane potentials (bottom row), for control (left), co-tuned (middle), and counter-tuned (right) population inactive. ***C***, CV_ISI_ and postsynaptic firing rate (mean and SD) in response to natural input for the 3 cases (top). Output firing rate as a function of the firing rate of the (compensatory) active inhibitory population (bottom). Arrowheads indicate the analyzed cases where output rate is equal to 5 Hz (i.e., where the green and purple lines cross the black line). ***D***, Pearson correlation between postsynaptic firing rate and excitatory input firing rates for different input signals for the three conditions in ***B***. Correlation between preferred (continuous line) or nonpreferred (dashed line) with the output activity as a function of the inhibitory firing rate of each inhibitory population (bottom). ***E***, Response to a pulse input in the phasic (left; first 50 ms) and tonic (right; last 50 ms) periods. Firing rate computed as the average number of spikes (for 100 trials) normalized by the bin size (50 ms). Each line corresponds to a different input strength; from light (low-amplitude pulse) to dark (high-amplitude pulse) colors. Insets, Tonic response for control firing rates.

Postsynaptic dynamics with two inhibitory populations with tuning that resulted from the combination of the Hebbian and the anti-Hebbian plasticity rules (Fig. 10*A*), that is, co-tuned and counter-tuned populations, were similar to that with co-tuned and flat inhibitory populations. In the unmodulated balanced state, output behavior is near identical to previous results (Fig. 10*B–D*, control). The main distinction between the models with counter-tuned or flat inhibitory profiles is how they complemented the co-tuned inhibitory currents: the flat inhibition produced currents that tracked the co-tuned inhibitory currents, whereas counter-tuned inhibition produced inhibitory currents that were largely uncorrelated to the co-tuned inhibitory currents (Fig. 10*B*, left; compare with Fig. 8*B*, left).

When either the co- or the counter-tuned inhibitory populations were inactivated, fluctuations in both firing rate and membrane potential increased considerably (Fig. 10*B*, middle and right). As before (Fig. 8), we adjusted the firing rate of the active inhibitory population so that the average output firing rate was 5 Hz in all conditions (Fig. 10*C*, top; crossing between green/purple line and the black horizontal line in Fig. 10*C*, bottom). Deactivation of the co-tuned population resulted in positive correlation between postsynaptic activity and preferred signals, and negative correlation between output and nonpreferred signals (Fig. 10*D*, purple). For transient stimulation, there was no discernible difference to the model with flat inhibition in the control state (Fig. 10*E*, top).

Turning off counter-tuned inhibition (Fig. 10*B–E*) also had similar results in the postsynaptic response as turning off the flat inhibition (compare Fig. 8*B–E*); that is, nonpreferred input produced output activity with positive correlation (Fig. 10*D*, green) and strong postsynaptic activity for transient activation (Fig. 10*E*, bottom). Unlike before, turning off co-tuned inhibition produced elevated firing rate responses also for transient stimuli from signals directly neighboring the preferred input (Fig. 10*E*, middle row, compare with Fig. 8*E*, middle).

### Quantitative differences of inhibitory profiles

For a better understanding of the differences between the three conditions studied here (one inhibitory population, co-tuned & flat, and co- & counter-tuned), we compared different modulation schemes quantitatively. We introduced the parameter $\Delta C=0.5(C_{\text{pref}}-C_{\text{non-pref}})$, that is, 50% of the difference in input/output correlation between preferred, *C*_pref,_ and nonpreferred, *C*_non-pref,_ signals (see Materials and Methods). Ideally, the sensory system should present three distinct responses for the three different modulatory conditions, which are captured by different values of Δ*C*. With unmodulated input (control), the output neuron should present uncorrelated activity with all input groups, and thus ΔC≈0. Modulated inputs (by decreasing the activity of either of the inhibitory populations) should correlate preferred (for one inhibitory inactive) and nonpreferred (for the other population inactive) to the output activity. This results in Δ*C* > 0 for correlated output/preferred signals, and Δ*C* < 0 for correlated output/nonpreferred signals.

**Figure 11.**
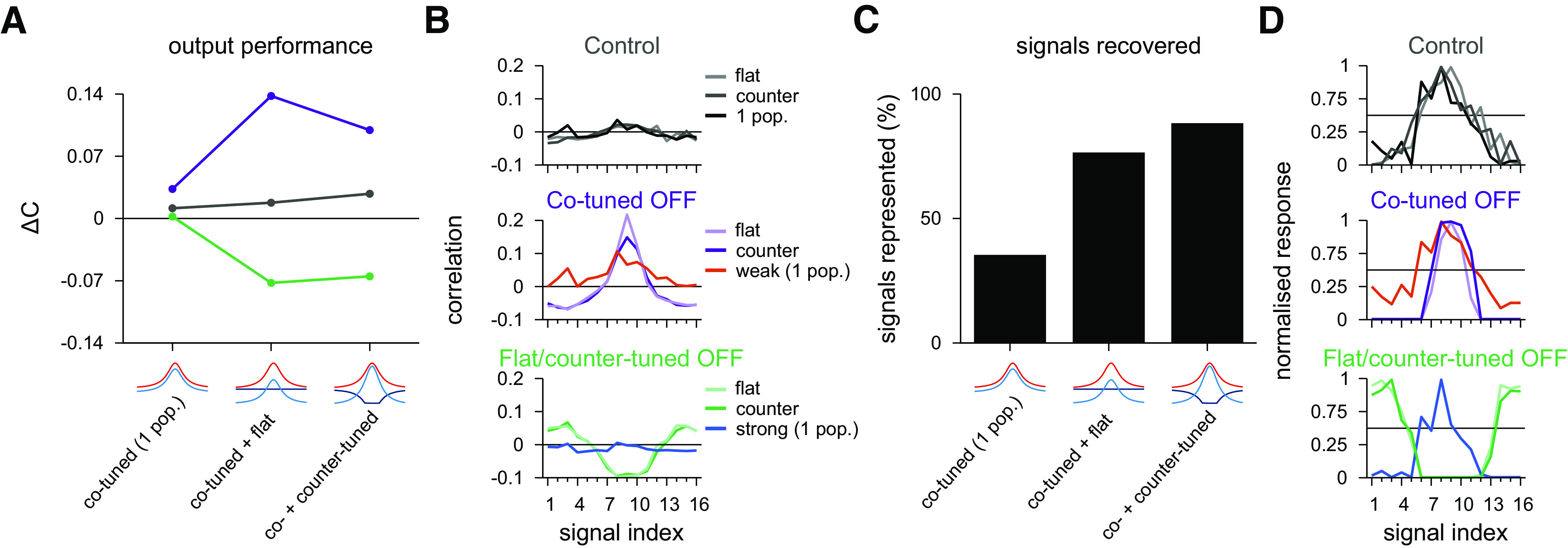
Comparison of postsynaptic responses receiving co-tuned & flat or co-tuned & counter-tuned inhibitory populations. ***A***, Performance index as the difference in input/output correlation between preferred and nonpreferred signal. Ideal outcome is Δ*C* = 0 for control case (gray), Δ*C* > 0 for co-tuned population inactive (purple), and Δ*C* < 0 for flat or counter-tuned populations inactive (green). We added the values for a single inhibitory population with control (gray), weak (purple), and strong (green) inhibitory inputs for comparison. ***B***, Pearson correlation between postsynaptic firing rate and excitatory input firing rates for different signal indices from Figures 4*D*, 8*D*, and 10*D*, replotted for reference. ***C***, Signals recovered in the pulse input paradigm. Signals represented are calculated as the percentage of signal afferents that activate the postsynaptic neuron with more than half the spikes of the maximum response for the 3 cases considered for the circuits analyzed together. ***D***, Normalized phasic response to a pulse input of 40 Hz, from Figures 4*E*, 8*E*, and 10*E*, replotted for reference. Horizontal line indicates 50% of maximum response.

In the control condition, we observed similar ΔC≈0 in all models (Fig. 11*A*, gray), reflecting low levels of correlation between output and input signals (Fig. 11*B*, top). With downregulated inhibition, Δ*C* increased slightly in the model with one homogeneous inhibitory population. Δ*C* increased more considerably in a two-population model in which the co-tuned population was inactive (Fig. 11*A*, purple), confirming an increased correlation between preferred signal and output (Fig. 11*B*, middle). When the flat or the counter-tuned inhibitory populations were inactivated, we observed postsynaptic responses even to nonpreferred input signals (Fig. 11*B*, bottom), which led to negative Δ*C* (Fig. 11*A*, green). Inactivating the flat inhibitory population resulted in a slightly better discrimination: larger positive Δ*C* (Fig. 11*A*, purple) and larger negative Δ*C* (Fig. 11*A*, green).

To compare pulse responses of the three models, we quantified which input signal groups elicited a substantial response to a pulse signal. We defined the number of signals recovered (Fig. 11*C*) as the number of responses with >50% of the maximum postsynaptic firing rate (Fig. 11*D*). The single inhibitory population model could only produce responses to preferred input signals, while co-modulation of two inhibitory populations could promote responses to nonpreferred input signals, as well. Counter-tuned population achieved better (i.e., broader) postsynaptic control than flat inhibition (Fig. 11*C*).

The addition of a second population of inhibitory inputs thus gives rise to a more flexible response to varying stimuli. In summary, our results shed light on the role of the many types of interneurons in cortical areas (Markram et al., 2004; Jiang et al., 2015; Gouwens et al., 2019), and show the benefits of combining different biologically inspired plasticity rules in neuronal networks.

## Discussion

We investigated how several distinctly tuned inhibitory connectivity profiles emerge through biologically reasonable plasticity rules and how they interact with a tuned excitatory connectivity profile in a receptive field-like paradigm. We found that the two aspects of selective attention (enhancing response to targets and suppressing the response to distractors) were implemented in our model by two types of disinhibition. Our results indicate a simple neuronal mechanism to help disentangle (or bind) parallel sensory input streams and may represent a step toward understanding the neural basis of intricate behaviors, such as the “cocktail party effect,” focusing on a single voice in a crowded, cacophonous place.

### Modulation of receptive field response

Our findings fit well with recent experimental results showing that pyramidal neurons in sensory areas of the cortex change their response to external stimuli depending on the context of the signal or attentional state (Bathellier et al., 2012; Ruff and Cohen, 2014, 2019; Benjamin et al., 2019; McClure and Polack, 2019). For example, principal neurons in macaque V4 respond to monochrome images of varying hues with variable response amplitude that is consistent with specific color tuning. However, the preferred color response of the neurons changes when naturally colored images are shown (Benjamin et al., 2019). In macaque V1, principal neurons can change the preferred orientation of visual stimuli when a pure tone is played alongside the visual stimulation (McClure and Polack, 2019). In the framework of our model, such a change in preference could be explained with differential input to the two inhibitory populations or by changes in their gains through contextual neuromodulation. Similarly, up to 20% of neurons in all areas of the mouse visual system (Billeh et al., 2019) were recently shown to change their preferred orientation according to the (spatial and temporal) frequency of the drifting gratings used in the experiments. In some cases, neuronal responses were shown to shift from their preferred to their nonpreferred input signals (a “flip” in response), similar to what we see in our simulations. These effects could also be explained by temporal fluctuations in the interaction of the two inhibitory populations, and the concurrent changes in transient responses of our model.

### Neuron types

The architecture of our model maps easily onto the neocortical microcircuit (Markram et al., 2004; Jiang et al., 2015). Co-tuned inhibition, for example, may originate from PV^+^ interneurons. As the main source of inhibition to pyramidal cells, PV^+^ interneurons target postsynaptic neurons with similar preferred orientation (Wilson et al., 2012), and activation of these neurons leads to broadened selectivity (Wilson et al., 2012; but see Lee et al., 2012). Flat or counter-tuned inhibition may arrive from SOM^+^ interneurons with their less selective connectivity patterns (Wilson et al., 2012). This interpretation is also in line with recent evidence suggesting that top-down visual attention relies on local inhibitory circuitry in primary visual cortex (Zhou et al., 2014). In this scheme, PV^+^ and SOM^+^ neurons inhibit pyramidal cells, whereas vasoactive intestinal peptide-positive neurons suppress other inhibitory interneurons, acting as a source of disinhibition. Direct manipulation of SOM^+^, PV^+^, and vasoactive intestinal peptide-positive neurons confirms these respective roles in inhibition and disinhibition in both visual (Fu et al., 2014) and auditory cortices (Pi et al., 2013; Kuchibhotla et al., 2017). Additionally, Zhou et al. (2014) reported that vasoactive intestinal peptide-positive neurons received excitatory top-down inputs from the rodent cingulate cortex, leading to a narrow selectivity profile of pyramidal cells when cingulate inputs are active and broad tuning when cingulate cortex is silent. This is analogous to deactivating the co-tuned population in our simulations. Finally, blocking cortical inhibition reduces the stimulus selectivity of cortical neurons (Sillito, 1979; Sillito et al., 1980; but see Nelson et al., 1994).

### Balance between excitatory and inhibitory inputs

In our model, we aimed for precise balance of excitation and inhibition, by way of a Hebbian-like inhibitory plasticity rule (Vogels et al., 2011), and accordant with evidence of excitatory and inhibitory co-tuning in cat visual cortex (Anderson et al., 2000), rodent auditory cortex (Wehr and Zador, 2003; Froemke et al., 2007; Zhou et al., 2014) and rodent hippocampus (Bhatia et al., 2019), and temporal correlations in neighboring excitatory and inhibitory synapses (Okun and Lampl, 2008). Consistent with earlier work, we could modulate the efficacy of a single inhibitory population to enhance the output correlation with the preferred input (Vogels and Abbott, 2009; Kremkow et al., 2010), but the flexibility of the control mechanism was very limited and nonpreferred signals never evoked faithful responses.

### Inhibitory synaptic plasticity

To explore how different inhibitory synaptic populations could form and interact, we split the inhibitory afferents into two populations and implemented a Hebbian-like inhibitory plasticity rule (Vogels et al., 2011; D'amour and Froemke, 2015) in one population that was coactive with either a homeostatic scaling (Stellwagen and Malenka, 2006; Zhong et al., 2018) or an anti-Hebbian (Woodin et al., 2003; Ormond and Woodin, 2009, 2011) plasticity rule. Woodin et al. (2003) recorded postsynaptic currents with the postsynaptic neuron clamped at a voltage below the reversal potential of chloride, effectively making a GABAergic synapse excitatory. Plasticity at those synapses changed the reversal potential of chloride toward a more depolarized value, making them stronger in those artificial conditions. However, once the voltage clamp is released, the inhibitory currents would be weaker than before learning because of the more depolarized chloride reversal potential that is closer to the neuron's resting membrane potential and thus exerts a smaller driving force (Hennequin et al., 2017, their Fig. 1*C*). These results suggest a plasticity rule that is Hebbian if the postsynaptic neuron's voltage is artificially kept below the reversal potential of chloride, but anti-Hebbian in normal conditions. The scaling plasticity rule acted locally but squeezed the distribution of all synaptic strengths to a narrow regimen, providing a parsimonious explanation for the untuned, blanket inhibition often encountered in experiments (Karnani et al., 2014), and providing easy means for modulating postsynaptic responses independently of the presynaptically tuned weight profiles. The anti-Hebbian rule was naturally unstable; that is, it could lead to infinite strengthening of weights and thus silent networks. Our implementation reinforces this outcome because inhibitory inputs are always active.

It is unclear how biological circuits would avoid such catastrophe, but in our model we could balance the effect of the two opposing rules and remain at plausible levels of postsynaptic activity by including a modulatory term that controlled the learning rate of the anti-Hebbian plasticity rule. While this mimics some of the observed modulatory control of plasticity through other neuronal types (Abraham and Bear, 1996; Froemke et al., 2007; Abs et al., 2018; Aljadeff et al., 2019), the reality is likely more complex, and possibly relies on finely orchestrated interaction of several different plasticity rules (Zenke et al., 2015; Hennequin et al., 2017), and their learning rates. Additionally, if the inhibitory neurons are driven laterally by excitatory neurons that lack excitatory recurrence, a form of anti-Hebbian plasticity is also stable (Hendin et al., 1997). No matter what form the ultimate mechanism may take, it is unlikely that it will affect the generality of our results.

### Parallels to artificial neural networks

Interestingly, artificial networks have been shown to develop similar receptive field profiles to the ones explored here when they are trained to solve multiple tasks (Yang et al., 2019). Yang et al. (2019) have shown that clusters of neurons can acquire co-tuned or flat connectivity, which are controlled by context-encoding signals. These results hint at the possibility that biological and artificial systems may use similar strategies to solve context-dependent filtering tasks.

### Additional biological complexity

To explore the interaction between two distinct inhibitory plasticity rules without confounds, we made the simplifying assumption that excitatory synapses would remain fixed (but see Litwin-Kumar and Doiron, 2014; Zenke et al., 2015; Clopath et al., 2016). Obviously, inhibitory plasticity rules do interact with multiple additional rules and constraints (e.g., excitatory or modulatory synaptic plasticity). Similarly, our model only considered a single postsynaptic neuron, with no feedback or lateral connectivity, which is thought to play an important role in cortical feature selectivity (Ahmed et al., 1994), and was theoretically shown to provide the means for multiplicative and additive modulation of receptive fields, and surround suppression (Litwin-Kumar et al., 2016). Other common features of sensory areas are the adaptation of postsynaptic responses to repetitive stimuli presentation (Kohn and Movshon, 2004) and the quick reshape of postsynaptic responses toward the attended stimuli (Fritz et al., 2003). These features may be explored in our model by including, for example, short-term plasticity (Tsodyks and Markram, 1997) in both inhibitory and excitatory synapses. Additional types of activity modulation may also reveal new features of networks with multiple types of inhibition and synaptic plasticity, such as induction of phase locking after inhibitory plasticity, as seen in mouse barrel cortex (Lourenço et al., 2020). Finally, other possible functions beyond simple input filtering, such as multiplexing or amplifying temporally varying signal streams (Naud and Sprekeler, 2018; Hertäg and Sprekeler, 2019), one-shot learning (Nicola and Clopath, 2019) must be considered. Our work only lays the groundwork for studies of multiple distinct plasticity rules in larger networks, with more complex excitatory-inhibitory interaction (Holmgren and Zilberter, 2001; Haas et al., 2006; Hennequin et al., 2017; Lourenço et al., 2020).

In conclusion, we predict that various GABAergic interneurons in the same cortical region must obey a range of different inhibitory synaptic plasticity rules, to restore or alter neuronal stimulus selectivity as appropriate and necessary. Such evidence would inform the theoretical framework presented here, and in turn inspire future computational modeling.
